# Supplementary material for: Global transcriptomic profiling and mutant analysis suggest linked 3-chlorobenzoate metabolism and activation of an integrative and conjugative element in Pseudomonas putida
Source: mSystems. 2026 Mar 23;11(4):e00024-26. doi: 10.1128/msystems.00024-26 (PMC13098234; doi:10.1128/msystems.00024-26)
Supplement: Supplemental material — Fig. S1-S3; Tables S1-S8. [file msystems.00024-26-s0001.pdf]

Supplementary material to:

Global transcriptomic profiling and mutant analysis suggest linked 3-chlorobenzoate metabolism and activation of an integrative and conjugative element in *Pseudomonas putida*

Roxane Bertholet*, Valentina Benigno*, Hanna Budny, Anthony Convers, Vladimir Sentchilo, Andrea Daveri, Nicolas Carraro and Jan Roelof van der Meer†

Department of Fundamental Microbiology, University of Lausanne, 1015 Lausanne, Switzerland

Fig. S1
Fig. S2
Fig. S3
Tables S1-S8

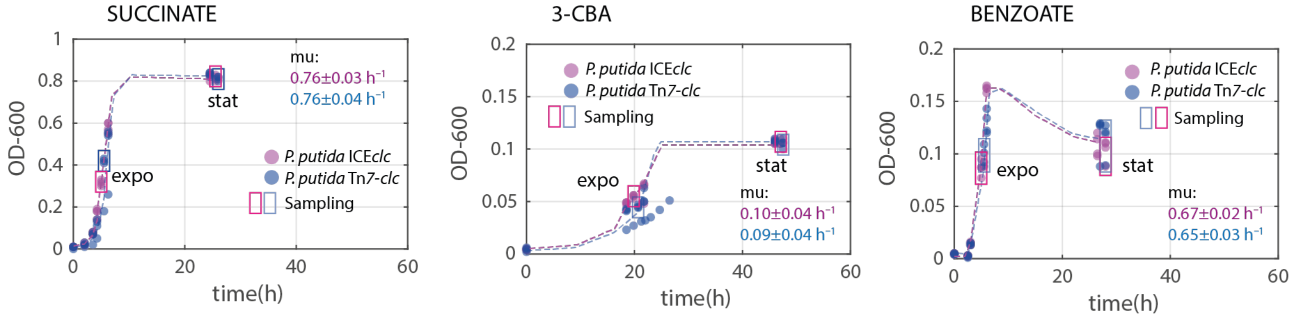

Supplementary Figure 1. Growth and sampling for RNAseq ('expo', 'stat') of quadruplicate cultures of *P. putida* 2737 (with ICE*clc*, magenta) and 3227 (mini-Tn*7*-*clc*, blue) on succinate, 3-CBA or benzoate.

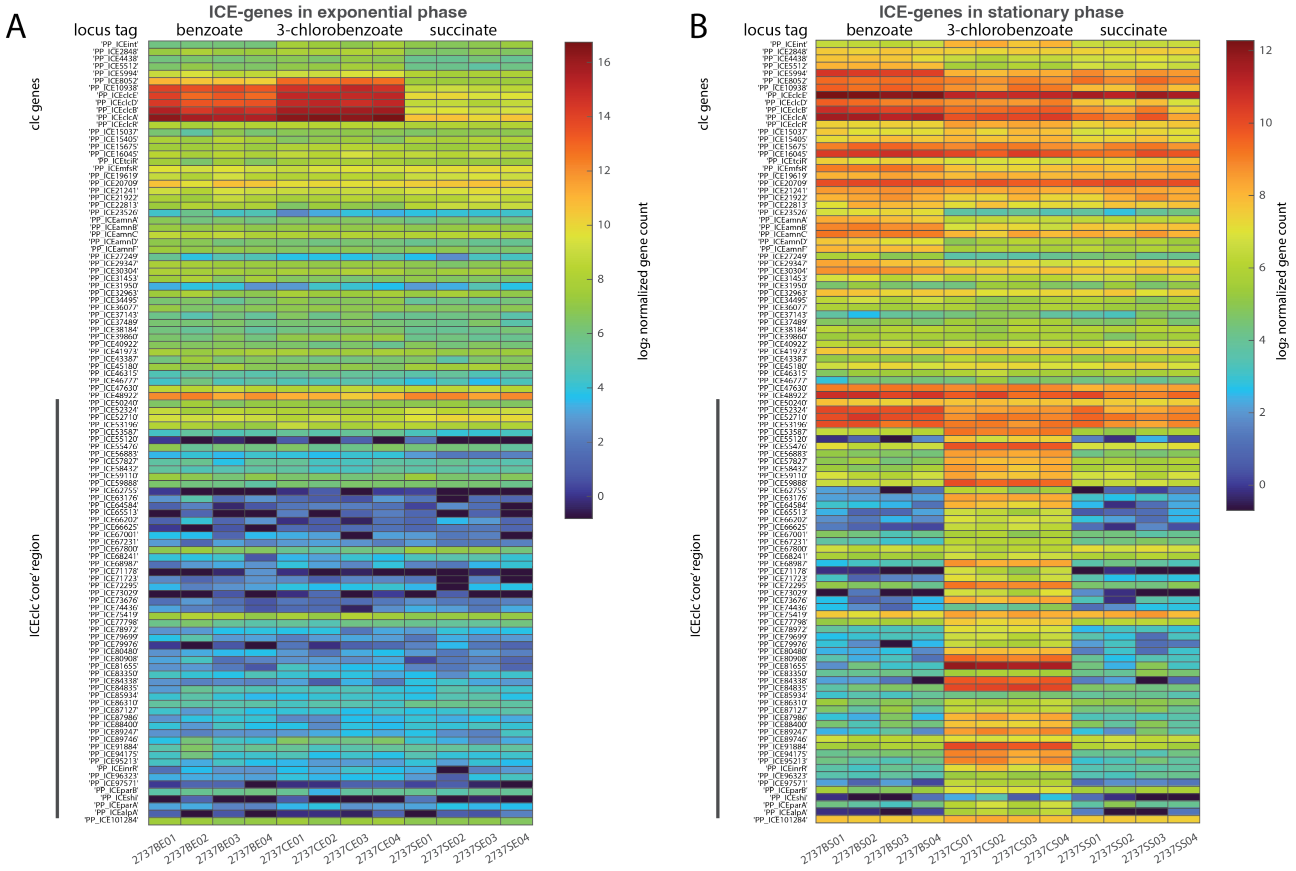

Supplementary Figure 2. Differential expression of ICE*clc* genes as a function of substrate and growth condition. A) Log_2-normalized gene count across four replicates of *P. putida* 2737 (with ICE*clc*) growing on benzoate, 3-chlorobenzoate or succinate in exponential phase (as in Fig. S1), or **B)** stationary phase. Locus tag of ICE-genes according to order of position on the ICE (direction from *attR* on top to *attL* on the bottom, as in Fig. 6), with the 'core'region indicated. Values represented by colors on the heatmap according to the scales on the right. Note the strong induction of the *clc*-genes during exponential growth on 3-chlorobenzoate but also their cross-induction during growth on benzoate. Also note the specific induction of most of the genes in the ICE core region during stationary phase on 3-chlorobenzoate, but not after growth on benzoate or succinate. Since this RNA isolated from the total population of cells, it cannot reflect that the higher expression of ICE core genes originates in only 3-5% of stationary phase cells.

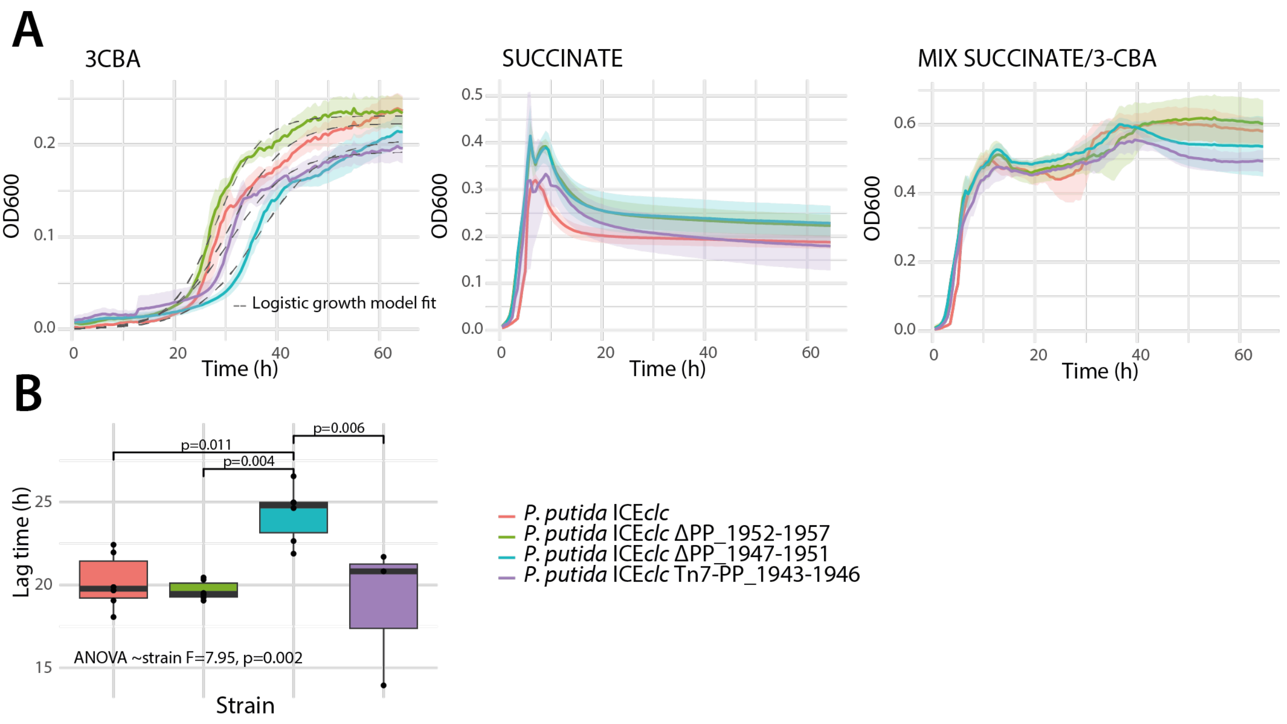

Supplementary Figure 3. A) Growth of triplicate cultures of *P. putida* 2737 (with ICE*clc*, red) and derived strains with ΔPP_1952-PP_1957 deletion, ΔPP_1947-PP_1851 deletion, and Tn7-PP_1943-PP_1946 on succinate, 3-CBA or a mix of succinate and 3-CBA. Coloured shades represent standard deviation. Logistic growth curves were fitted individually using nonlinear least-squares fitting (dashed lines). **B)** Lag time for each strain during growth on 3-CBA. Lag time was calculated from the fitted logistic parameters. ANOVA was used to evaluate lag times differences between groups. Post hoc comparisons were performed using Tukey's Honestly Significant Difference test (individual significant adjusted p-values indicated).

Supplementary Table 1. Strains used in this study.

Strain number	Strain	Description	Reference
Pseudomonas putida			
2737	UWC1 ICE*clc* *tRNA-Gly* 5	Plasmid-free derivative of *P. putida* KT2440, rifampicin resistant, containing 1 copy ICE*clc* at *tRNA-Gly* 5	(1)
3227	UWC1 *clc* operon	Plasmid-free derivative of *P. putida* KT2440, rifampicin resistant, containing the *clcRABDE* operon into the attTn7 site on the chromosome	(2)
7262	UWC1 ICE*clc* *tRNA-Gly* 5 Δ*clcRABDE* operon pJP-*amnB* -Km	Strain 2737 with a deletion of the *clc* operon from ICE*clc,* transformed with pJP-*amnB* -Km	This study
7434	UWC1 ICE*clc* *tRNA-Gly* 5, Δ*benABCD* , pJP-*amnB* -Km,	Strain 2737 with a deletion of the *benABCD* operon from the chromosome, transformed with pJP-*amnB* -Km	This study
7260	UWC1 ICE*clc* *tRNA-Gly* 5 pJP-*amnB* -Km	Strain 2737 transformed with pJP-*amnB* -Km	This study
7301	UWC1 ICE*clc* *tRNA-Gly* 5 *ΔclcRABDE* pJP-*amnB* -Km, Tn7::*clcRABDE*	Strain 7262 with complementation of the *clcRABDE* operon via pUCminiTn7 insertion	This study
7437	UWC1 ICE*clc* *tRNA-Gly* 5 *ΔclcRABDE* pJP-*amnB* -Km, Tn7::*clcR*	Strain 7262 with complementation of *clcR* via pUCminiTn7 insertion	This study
7438	UWC1 ICE*clc* *tRNA-Gly* 5 *ΔclcRABDE* pJP-*amnB* -Km, Tn7::*clcRAB*	Strain 7262 with complementation of *clcRAB* via pUCminiTn7 insertion	This study
7439	UWC1 ICE*clc* *tRNA-Gly* 5 *ΔclcRABDE* pJP-*amnB* -Km, Tn7::*clcRA*	Strain 7262 with complementation of *clcRA* via pUCminiTn7 insertion	This study
7016	UWC1 ICE*clc* *tRNA-Gly* 5, Tn5::P*inR-echerry*	Strain 2737 transformed with with pTnMOd-oTC::P*inR-echerry*	This study
6987	UWC1 ICE*clc* *tRNA-Gly* 5 *ΔclcRABDE* , Tn5::P*inR-echerry*	Strain 2737 with a deletion of the *clc* operon from ICE*clc* , transformed with with pTnMOd-oTC::P*inR-echerry*	This study
7472	UWC1 ICE*clc* *tRNA-Gly* 5, Δ*benABCD* , Tn5::P*inR-echerry*	Strain 2737 with a deletion of the *benABCD* operon from the chromosome, transformed with with pTnMOd-oTC::P*inR-echerry*	This study
7527	UWC1 ICE*clc* *tRNA-Gly* 5, Δorf8052	Strain 2737 with a deletion of orf8052 from the ICE*clc* element	This study
7525	UWC1 ICE*clc* *tRNA-Gly* 5, Δorf5994-5512	Strain 2737 with a deletion of orfs 5994-5512 from the ICE*clc* element	This study
7784	UWC1 ICE*clc* *tRNA-Gly* 5, Δorf5994-5512 Tn5::P*inR-echerry*	Strain 7525 transformed with pTnMOd-oTC::P*inR-echerry*	This study
7789	UWC1 ICE*clc* *tRNA-Gly* 5, Δorf8052 Tn5::P*inR-echerry*	Strain 7527 transformed with pTnMOd-oTC::P*inR-echerry*	This study
8080	UWC1 ICE*clc* *tRNA-Gly* 5 Tn7::Pl*ac* -PP_1943-PP_1946	Strain 2737 transformed with pUC18-miniTn7T-Lac vector for IPTG-controlled expression of genes PP_1943 to PP_1946	This study
8059	UWC1 ICE*clc* *tRNA-Gly* 5 ΔPP_1952-PP_1957	Strain 2737 with a deletion of genes PP_1952 to PP_1957	This study
8061	UWC1 ICE*clc* *tRNA-Gly* 5 ΔPP_1947-PP_1951	Strain 2737 with a deletion of genes PP_1947 to PP_1951	This study
8100	UWC1 ICE*clc* *tRNA-Gly* 5 Tn7::PP_1943-PP_1946 Tn5::P*inR-echerry*	Strain 8080 transformed with plasmid pCK218::P*inR-echerry*	This study
8062	UWC1 ICE*clc* *tRNA-Gly* 5 ΔPP_1952-PP_1957 Tn5::P*inR-echerry*	Strain 8059 transformed with plasmid pCK218::P*inR-echerry*	This study
8064	UWC1 ICE*clc* *tRNA-Gly* 5 ΔPP_1947-PP_1951 Tn5::P*inR-echerry*	Strain 8061 transformed with plasmid pCK218::P*inR-echerry*	This study
2744	UWCGC Tn7::P*tac* -*mcherry* -Gm	*P. putida* UWCGC carrying Tn7::P*tac-mcherry* (used as ICE*clc* recipient)	(3)
3488	UWC1 Tn5	*P. putida* UWC1 carrying pTnMOd-oTC (used as ICE*clc* recipient)	Nicolas Pradervand
Escherichia coli			
1625	SM10/λPir pUX-BF13	Tn7 helper plasmid	(4)
3044	DH5aλPir	Host for plasmids propagation with R6K origin of replication, λpir lysogen	Victor de Lorenzo
2740	DH5aλPir pUC18-miniTn7T-Lac	GmR, AmpR, mini Tn7 system	(5)
2917	DH5aλPir pEMG	KmR, oriR6K, Ptac-lacZa, I-SceI sites	(6)
2918	DH5aλPir pSW	AmpR, oriRK2, Pm-I-SceI	(6)
3618	DH5aλPir pTnMOd-oTC::PinR-echerry	pTnMod-OTc miniTn5 carrying in its NotI site P*inR-echerry*	Ryo Miyazaki
3154	DH5aλPir pCK218::PinR-echerry	pCK218 miniTn5 with fusion P*inR-echerry*	Marco Minoia

1. Sentchilo V, Czechowska K, Pradervand N, Minoia M, Miyazaki R, van der Meer JR. 2009. Intracellular excision and reintegration dynamics of the ICE*clc* genomic island of Pseudomonas knackmussii sp. strain B13. Molecular Microbiology 72:1293–1306.
2. Reinhard F, Miyazaki R, Pradervand N, van der Meer JR. 2013. Cell differentiation to “mating bodies” induced by an Integrating and Conjugative Element in free-living bacteria. Current Biology 23:255–259.
3. Miyazaki R, van der Meer JR. 2011. A dual functional origin of transfer in the ICE*clc* genomic island of *Pseudomonas knackmussii* B13: *oriT* of ICE*clc* . Molecular Microbiology 79:743–758.
4. Koch B, Jensen LE, Nybroe O. 2001. A panel of Tn7-based vectors for insertion of the *gfp* marker gene or for delivery of cloned DNA into Gram-negative bacteria at a neutral chromosomal site. Journal of Microbiological Methods 45:187–195.
5. Choi K-H, Gaynor JB, White KG, Lopez C, Bosio CM, Karkhoff-Schweizer RR, Schweizer HP. 2005. A Tn7-based broad-range bacterial cloning and expression system. Nat Methods 2:443–448.
6. Martínez-García E, de Lorenzo V. 2011. Engineering multiple genomic deletions in Gram-negative bacteria: analysis of the multi-resistant antibiotic profile of Pseudomonas putida KT2440: Tools for editing Gram-negative genomes. Environmental Microbiology 13:2702–2716.

Supplementary Table 2. Commonly differentially expressed genes among exponential-stationary phase comparisons.

common higher in expo (upstruct)	common higher in stat (downstruct)	common higher in expo (upstruct)	common higher in stat (downstruct)	common higher in expo (upstruct)	common higher in stat (downstruct)	common higher in expo (upstruct)	common higher in stat (downstruct)
PP_0006	PP_0201	PP_0814	PP_2685	PP_1944	PP_3231	PP_4588	PP_4761
PP_0011	PP_0411	PP_0816	PP_2688	PP_1950	PP_3312	PP_4708	PP_5121
PP_0013	PP_0537	PP_0834	PP_2690	PP_1954	PP_3315	PP_4742	PP_5122
PP_0058	PP_0562	PP_0836	PP_2708	PP_1957	PP_3316	PP_4795	
PP_0060	PP_0668	PP_0897	PP_2709	PP_2089	PP_3318	PP_4800	
PP_0082	PP_0683	PP_0915	PP_2710	PP_2439	PP_3396	PP_4803	
PP_0113	PP_1083	PP_0975	PP_2711	PP_2453	PP_3398	PP_4828	
PP_0178	PP_1147	PP_0989	PP_2712	PP_2474	PP_3400	PP_4861	
PP_0225	PP_1410	PP_0998	PP_2720	PP_3106	PP_3401	PP_4891	
PP_0226	PP_1449	PP_1001	PP_2723	PP_3108	PP_3422	PP_4892	
PP_0227	PP_1511	PP_1002	PP_2725	PP_3154	PP_3423	PP_4940	
PP_0234	PP_1516	PP_1003	PP_2726	PP_3328	PP_3472	PP_4941	
PP_0235	PP_1528	PP_1068	PP_2727	PP_3343	PP_3476	PP_4942	
PP_0268	PP_1633	PP_1069	PP_2728	PP_3496	PP_3477	PP_4946	
PP_0282	PP_1651	PP_1070	PP_2729	PP_3593	PP_3478	PP_5022	
PP_0300	PP_1652	PP_1071	PP_2730	PP_3676	PP_3479	PP_5023	
PP_0338	PP_1748	PP_1084	PP_2731	PP_3706	PP_3481	PP_5024	
PP_0339	PP_1828	PP_1116	PP_2732	PP_3707	PP_3482	PP_5053	
PP_0403	PP_1849	PP_1128	PP_2735	PP_3720	PP_3483	PP_5075	
PP_0421	PP_1937	PP_1179	PP_2740	PP_3821	PP_3490	PP_5155	
PP_0445	PP_1962	PP_1206	PP_2751	PP_3904	PP_3519	PP_5156	
PP_0448	PP_1963	PP_1210	PP_2754	PP_3988	PP_3552	PP_5165	
PP_0451	PP_1964	PP_1213	PP_2755	PP_4012	PP_3556	PP_5171	
PP_0452	PP_1966	PP_1219	PP_2757	PP_4094	PP_3576	PP_5172	
PP_0454	PP_1967	PP_1222	PP_2758	PP_4123	PP_3698	PP_5177	
PP_0455	PP_1970	PP_1223	PP_2764	PP_4124	PP_3794	PP_5178	
PP_0457	PP_1971	PP_1240	PP_2774	PP_4126	PP_3815	PP_5179	
PP_0460	PP_2006	PP_1245	PP_2803	PP_4127	PP_4210	PP_5181	
PP_0461	PP_2038	PP_1247	PP_2808	PP_4128	PP_4302	PP_5188	
PP_0463	PP_2047	PP_1272	PP_2818	PP_4129	PP_4328	PP_5193	
PP_0464	PP_2061	PP_1303	PP_2830	PP_4130	PP_4334	PP_5238	
PP_0466	PP_2090	PP_1304	PP_2834	PP_4131	PP_4341	PP_5335	
PP_0470	PP_2118	PP_1318	PP_2840	PP_4149	PP_4344	PP_5338	
PP_0471	PP_2185	PP_1319	PP_2855	PP_4178	PP_4361	PP_5366	
PP_0474	PP_2186	PP_1361	PP_2856	PP_4179	PP_4369	PP_5377	
PP_0478	PP_2192	PP_1366	PP_2857	PP_4185	PP_4371	PP_5378	
PP_0479	PP_2232	PP_1377	PP_2858	PP_4186	PP_4372	PP_5379	
PP_0480	PP_2235	PP_1430	PP_2869	PP_4187	PP_4382	PP_5380	
PP_0558	PP_2236	PP_1496	PP_2876	PP_4188	PP_4383	PP_5412	
PP_0559	PP_2237	PP_1599	PP_2878	PP_4189	PP_4384	PP_5413	
PP_0570	PP_2270	PP_1600	PP_2885	PP_4190	PP_4386	PP_5414	
PP_0596	PP_2344	PP_1601	PP_2887	PP_4191	PP_4387	PP_5415	
PP_0606	PP_2385	PP_1605	PP_2896	PP_4192	PP_4391	PP_5416	
PP_0616	PP_2429	PP_1612	PP_2897	PP_4206	PP_4394	PP_5417	
PP_0617	PP_2508	PP_1617	PP_2972	PP_4276	PP_4396	PP_5418	
PP_0618	PP_2514	PP_1689	PP_3010	PP_4305	PP_4406	PP_5419	
PP_0619	PP_2517	PP_1714	PP_3014	PP_4308	PP_4407	PP_ICEclcA	
PP_0620	PP_2542	PP_1787	PP_3015	PP_4409	PP_4521	PP_ICEclcD	
PP_0658	PP_2561	PP_1789	PP_3016	PP_4410	PP_4523		
PP_0674	PP_2586	PP_1790	PP_3061	PP_4435	PP_4524		
PP_0693	PP_2594	PP_1791	PP_3062	PP_4448	PP_4561		
PP_0694	PP_2623	PP_1792	PP_3153	PP_4486	PP_4612		
PP_0721	PP_2655	PP_1794	PP_3175	PP_4519	PP_4619		
PP_0799	PP_2658	PP_1943	PP_3183	PP_4541	PP_4631		

Supplementary Table 3. Differentially expressed host genes in *P. putida* with or without ICE*clc.*

Lower in 2737

Gene ID	p-value	Log2 fold change	Gene name	Description
PP_0211	0.000256253	-2.492881461	-	hypothetical protein PP_0211
PP_0723	0.023669649	-3.621449688	*ipk*	4-diphosphocytidyl-2-C-methyl-D-erythritol kinase
PP_0765	0.034512232	-2.433297086	-	hypothetical protein PP_0765
PP_0766	0.026343681	-2.259420733	-	hypothetical protein PP_0766
PP_1110	0.034483918	-2.367788642	-	serine O-acetyltransferase
PP_1111	0.049722708	-2.37851859	-	synthetase
PP_1112	0.040419077	-2.422217035	-	major facilitator superfamily protein
PP_1170	0.023630644	-2.024989231	-	SMP-30/gluconolaconase/LRE domain-containing protein
PP_1410	4.42E-07	-3.518959199	-	hypothetical protein PP_1410
PP_1516	0.00121848	-2.872976272	-	RND family efflux transporter MFP subunit
PP_1517	0.000338804	-2.552002347	-	acriflavin resistance protein
PP_2472	9.30E-07	-2.593688934	-	hypothetical protein PP_2472
PP_2520	0.002135284	-2.555253021	-	hypothetical protein PP_2520
PP_2734	2.04E-06	-30.35021535	*cfa*	cyclopropane-fatty-acyl-phospholipid synthase
PP_3407	7.28E-06	-846.2153296	-	hypothetical protein PP_3407
PP_3863	0.039977085	-2.100047642	-	phage tail protein
PP_3871	0.031756098	-2.609822152	-	hypothetical protein PP_3871
PP_3873	0.045144844	-2.347271125	-	hypothetical protein PP_3873
PP_3874	0.0421353	-2.179837829	-	hypothetical protein PP_3874
PP_3877	0.04891815	-2.311812928	-	hypothetical protein PP_3877
PP_3921	0.010873956	-42.62840802	-	hypothetical protein PP_3921
PP_3992	0.000572551	-5.140034003	-	xanthine/uracil permease
PP_4040	0.000309589	-2.206197117	-	glyoxalase/bleomycin resistance protein/dioxygenase
PP_4321	0.032475787	-2.642795229	*dsbE*	thiol--disulfide oxidoreductase

Higher in 2737

Gene ID	p-value	Log2 fold change	Gene name	Description
PP_0086	0.013910782	2.237645702	-	hypothetical protein PP_0086
PP_2639	0.035646335	2.282232062	-	dihydrodipicolinate synthase
PP_2964	8.49E-07	4.730478074	*tnpA*	Tn4652, transposase
PP_2965	3.64E-06	2.556030345	-	Tn4652, tnpA repressor protein TnpC
PP_2966	6.25E-05	2.031873235	-	hypothetical protein PP_2966
PP_2973	0.022962307	2.224999808	*dgkA-2*	diacylglycerol kinase
PP_2975	2.90E-06	2.185791951	-	transposase
PP_2980	4.58E-05	2.09361614	-	hypothetical protein PP_2980
PP_5173	3.97E-06	3.477188826	-	acriflavin resistance protein
PP_5174	4.85E-07	2.839939449	-	RND efflux transporter
PP_5175	2.61E-06	4.831432881	-	RND family efflux transporter MFP subunit

Supplementary Table 4. Differentially expressed genes in exponential phase during growth on 3-CBA or benzoate.

Higher on 3-CBA

Gene ID	baseMean (normalized)	log2 Fold Change	p- value	KO term	Description
PP_3622	306.04	-18.47	2.34E-06	K07303	isoquinoline 1-oxidoreductase subunit beta
PP_2096	282.81	-3.62	4.35E-05	K12297	23S rRNA m(2)G2445 methyltransferase
PP_4050	655.50	-4.39	1.25E-04	K00703	glycogen synthase
PP_0581	224.34	-3.17	1.46E-04	K00059	3-ketoacyl-ACP reductase
PP_2264	354.92	-13.44	2.41E-04	K17321	ABC transporter substrate-binding protein
PP_4053	245.38	-6.81	2.52E-04	K06044	malto-oligosyltrehalose synthase
PP_2344	196.56	-2.95	2.57E-04		hypothetical protein PP_2344
PP_0255	217.28	-4.91	3.02E-04		hypothetical protein PP_0255
PP_0711	127.53	-17.45	3.39E-04		isochorismatase superfamily hydrolase
PP_1893	754.16	-3.63	3.92E-04	K06445	acyl-CoA dehydrogenase
PP_2259	69.97	-5.18	4.28E-04	K21405	Fis family transcriptional regulator
PP_4651	206.94	-6.06	4.41E-04	K00425	ubiquinol oxidase subunit I. cyanide insensitive
PP_1948	88.12	-591.96	4.58E-04		benzaldehyde dehydrogenase
PP_5173	1730.01	-2.63	4.75E-04	K21134	acriflavin resistance protein
PP_3425	59.96	-8.40	5.13E-04	K18298	RND family efflux transporter MFP subunit
PP_2475	871.02	-3.99	5.28E-04	K16137	TetR family transcriptional regulator
PP_2348	81.47	-3.03	5.36E-04		integral membrane sensor signal transduction histidine kinase
PP_4685	1853.71	-4.09	5.44E-04	K07343	hypothetical protein PP_4685
PP_1482	82.85	-3.21	5.61E-04	K02053	binding-protein-dependent transport system inner membrane protein
PP_2995	88.65	-2.48	5.67E-04		DNA topology modulation kinase FlaR
PP_1944	891.70	-126.33	5.77E-04		glycine cleavage system protein T
PP_4282	191.09	-12.59	5.99E-04	K06188	aquaporin Z
PP_3426	195.64	-7.97	6.04E-04	K18299	hydrophobe/amphiphile efflux-1 (HAE1) family transporter
PP_1484	122.61	-3.07	6.11E-04	K02052	spermidine/putrescine ABC transporter ATPase
PP_2563	183.29	-7.35	6.17E-04		antibiotic biosynthesis protein
PP_3668	1824.34	-4.10	6.21E-04	K03782	catalase/peroxidase HPI
PP_4054	215.41	-6.82	6.33E-04		hypothetical protein PP_4054
PP_4111	708.71	-3.24	6.56E-04	K02355	elongation factor G
PP_3558	333.07	-6.29	6.60E-04	K02002	glycine betaine ABC transporter substrate-binding protein
PP_1196	563.10	-2.03	6.69E-04		hypothetical protein PP_1196
PP_0582	344.15	-2.95	6.74E-04	K00626	acetyl-CoA acetyltransferase
PP_2558	42.28	-5.51	6.79E-04	K12538	TolC family type I secretion outer membrane protein
PP_2797	72.74	-22.28	7.01E-04	K14393	acetate permease
PP_2741	204.40	-2.79	7.07E-04		mechanosensitive ion channel protein MscS
PP_0777	725.09	-2.97	7.43E-04	K00432	glutathione peroxidase
PP_3566	549.70	-14.61	7.43E-04		major facilitator family transporter
PP_4011	2624.83	-3.43	7.64E-04	K00031	isocitrate dehydrogenase
PP_2856	125.86	-3.57	7.80E-04		hypothetical protein PP_2856
PP_4565	355.35	-2.75	7.82E-04	K00906	bifunctional isocitrate dehydrogenase kinase/phosphatase
PP_1931	279.48	-3.65	8.30E-04		hypothetical protein PP_1931
PP_3545	184.80	-3.98	8.35E-04		PAS/PAC sensor hybrid histidine kinase
PP_3955	325.74	-3.69	8.61E-04		transporter
PP_1444	767.03	-2.36	8.92E-04	K00117	glucose dehydrogenase
PP_1684	673.13	-8.31	9.18E-04		major facilitator superfamily transporter
PP_2195	82.36	-2.28	9.25E-04	K11073	polyamine ABC transporter substrate-binding protein
PP_3623	215.97	-17.49	9.34E-04		gluconate 2-dehydrogenase
PP_4554	38.34	-5.02	9.59E-04		activator of Hsp90 ATPase 1 family protein
PP_2795	57.17	-19.90	9.70E-04	K00666	acyl-CoA synthetase
PP_1983	459.56	-3.21	9.82E-04		sensory box protein
PP_1953	257.76	-14.34	9.88E-04		short chain dehydrogenase/reductase oxidoreductase
PP_1952	115.21	-87.08	1.03E-03		metallo-beta-lactamase
PP_3247	53.51	-3.98	1.04E-03	K03453	bile acid/Na+ symporter family protein
PP_4033	146.29	-17.70	1.06E-03	K00784	ribonuclease Z
PP_4052	328.27	-4.07	1.07E-03	K00705	4-alpha-glucanotransferase
PP_4650	121.95	-5.99	1.08E-03	K00426	cytochrome d ubiquinol oxidase subunit II
PP_3138	327.71	-5.14	1.08E-03		VirK domain-containing protein
PP_4038	2260.89	-6.41	1.08E-03	K17723	dihydropyrimidine dehydrogenase
PP_4553	214.74	-3.10	1.08E-03	K03088	ECF subfamily RNA polymerase sigma factor
PP_4555	56.27	-4.87	1.09E-03		DGPFAETKE family protein
PP_1408	521.31	-3.00	1.09E-03		alpha/beta hydrolase
PP_3135	861.44	-4.02	1.12E-03		glycosyl transferase family protein
PP_3692	1368.65	-2.32	1.12E-03		hypothetical protein PP_3692
PP_1415	37.39	-2.46	1.12E-03	K07120	hypothetical protein PP_1415
PP_3137	320.08	-4.31	1.14E-03		glycosyl transferase family protein
PP_5274	266.70	-239.43	1.16E-03		hypothetical protein PP_5274
PP_1943	838.31	-94.56	1.19E-03	K01433	formyltetrahydrofolate deformylase
PP_3402	65.96	-10.40	1.19E-03		hypothetical protein PP_3402
PP_1483	82.14	-4.00	1.21E-03	K02054	binding-protein-dependent transport system inner membrane protein
PP_2714	237.58	-2.36	1.23E-03	K07645	sensor histidine kinase
PP_5252	176.13	-3.35	1.24E-03		amidohydrolase
PP_2262	22.14	-16.58	1.25E-03	K17322	binding-protein-dependent transport system inner membrane protein
PP_3548	145.36	-4.13	1.27E-03	K03446	EmrB/QacA family drug resistance transporter
PP_3621	390.25	-20.24	1.29E-03	K07302	(2Fe-2S)-binding protein
PP_4258	1290.91	-3.59	1.30E-03	K00406	cytochrome c oxidase. cbb3-type subunit III
PP_3140	416.58	-4.32	1.31E-03		glycosyl transferase family protein
PP_2925	216.27	-7.93	1.32E-03	K00116	malate:quinone oxidoreductase
PP_2320	1013.50	-2.60	1.33E-03	K16291	ErfK/YbiS/YcfS/YnhG family protein
PP_2697	47.95	-3.28	1.35E-03		flavin reductase domain-containing protein
PP_0906	302.94	-4.19	1.36E-03	K18138	multidrug efflux protein
PP_1122	270.75	-3.19	1.38E-03		OmpA/MotB domain-containing protein
PP_3690	497.21	-2.30	1.38E-03		hypothetical protein PP_3690
PP_3419	370.16	-3.92	1.39E-03		Fis family transcriptional regulator
PP_1950	181.45	-335.52	1.40E-03	K22553	hypothetical protein PP_1950
PP_4034	957.11	-6.24	1.40E-03	K06016	allantoate amidohydrolase
PP_1949	137.39	-216.76	1.41E-03		GMC family oxidoreductase
PP_1954	104.59	-23.35	1.41E-03		hypothetical protein PP_1954
PP_2853	892.30	-3.95	1.43E-03		hypothetical protein PP_2853
PP_1557	16.90	-8.40	1.43E-03		hypothetical protein PP_1557
PP_1532	105.48	-7.96	1.43E-03		phage integrase
PP_2505	351.52	-3.36	1.44E-03		GAF domain/GGDEF domain-containing protein
PP_1955	148.38	-20.81	1.47E-03		cytochrome P450 family protein
PP_4449	183.36	-2.20	1.47E-03		hypothetical protein PP_4449
PP_4496	166.31	-3.61	1.48E-03	K11734	amino acid ABC transporter permease
PP_1581	255.36	-5.64	1.49E-03		BNR repeat-containing protein
PP_0715	121.70	-3.41	1.49E-03		NodT family RND efflux system outer membrane lipoprotein
PP_1569	15.86	-7.90	1.49E-03		hypothetical protein PP_1569
PP_2850	2.06	-9.77	1.51E-03		hypothetical protein PP_2850
PP_2135	4.94	-4.08	1.52E-03		hypothetical protein PP_2135
PP_1215	82.98	-2.02	1.52E-03	K01159	Holliday junction resolvase
PP_1126	492.13	-2.10	1.53E-03		Beta-agarase
PP_0920	511.78	-4.42	1.56E-03		NADH:flavin oxidoreductase
PP_3430	715.42	-2.52	1.57E-03		PAS/PAC sensor hybrid histidine kinase
PP_2363	156.73	-4.08	1.57E-03		spore coat U domain-containing protein
PP_1761	1412.02	-2.38	1.58E-03		sensory box protein/GGDEF family protein
PP_3611	277.61	-4.78	1.58E-03		hypothetical protein PP_3611
PP_3942	20.58	-4.78	1.59E-03	K01799	maleate cis-trans isomerase
PP_0976	53.16	-2.37	1.60E-03	K06970	SAM-dependent methyltransferase
PP_2483	62.98	-2.77	1.60E-03	K07141	hypothetical protein PP_2483
PP_2796	10.89	-40.75	1.61E-03		hypothetical protein PP_2796
PP_2705	112.70	-3.67	1.63E-03		hypothetical protein PP_2705
PP_4820	800.82	-2.46	1.63E-03	K05540	NifR3 family TIM-barrel protein
PP_2852	867.82	-2.49	1.63E-03		sulfatase
PP_4059	289.50	-2.86	1.64E-03	K05343	trehalose synthase
PP_4041	339.18	-2.17	1.66E-03		glycoside hydrolase 15-like protein
PP_1494	730.61	-2.53	1.69E-03	K11444	response regulator/GGDEF domain-containing protein
PP_1486	921.85	-2.83	1.69E-03	K02055	polyamine ABC transporter substrate-binding protein
PP_3683	151.04	-3.15	1.70E-03		hypothetical protein PP_3683
PP_4255	1170.99	-3.19	1.70E-03	K00404	cbb3-type cytochrome c oxidase subunit I
PP_2312	422.87	-2.88	1.76E-03		lytic transglycosylase
PP_3448	246.46	-5.15	1.79E-03	K11740	bacteriophage N4 adsorption protein B
PP_2124	123.29	-4.71	1.79E-03	K21001	glycosyl transferase family protein
PP_0109	37.65	-20.60	1.80E-03	K02259	cytochrome oxidase assembly
PP_4021	144.43	-5.93	1.82E-03	K00433	alpha/beta hydrolase
PP_3559	47.17	-7.17	1.85E-03	K02001	binding-protein-dependent transport system inner membrane protein
PP_3141	147.64	-4.87	1.88E-03		WecB/TagA/CpsF family glycosyl transferase
PP_1144	405.16	-14.32	1.89E-03		diguanylate cyclase
PP_0105	151.27	-4.64	1.90E-03	K02258	cytochrome C oxidase assembly protein
PP_3130	557.49	-2.77	1.91E-03		hypothetical protein PP_3130
PP_ICE8052	1594.01	-4.36	1.91E-03	K02116	PP_aacC1
PP_ICEclcE	10836.61	-3.20	1.92E-03		PP_aacC1
PP_2308	536.78	-2.85	1.93E-03		acyl-CoA thioesterase
PP_3431	1360.35	-3.74	1.95E-03		ThiJ/PfpI domain-containing protein
PP_1862	75.53	-4.91	1.98E-03	K07090	hypothetical protein PP_1862
PP_2798	41.13	-2.61	1.99E-03		oxidoreductase
PP_4035	1296.23	-6.84	1.99E-03	K03457	NCS1 nucleoside transporter
PP_4702	575.90	-2.67	2.00E-03	K01895	acetyl-CoA synthetase
PP_2292	1536.49	-5.23	2.00E-03		hypothetical protein PP_2292
PP_1584	73.30	-5.00	2.06E-03		hypothetical protein PP_1584
PP_2696	23.78	-7.83	2.08E-03		transcriptional activator MetR
PP_1152	205.62	-2.53	2.08E-03		secretion protein HlyD family protein
PP_0589	281.14	-2.98	2.09E-03	K07552	Bcr/CflA family multidrug resistance transporter
PP_2056	69.18	-4.10	2.09E-03	K11103	sodium:dicarboxylate symporter
PP_2386	130.71	-2.08	2.10E-03		hypothetical protein PP_2386
PP_4055	422.06	-4.12	2.11E-03	K01214	glycogen debranching protein GlgX
PP_2551	53.97	-3.26	2.14E-03		LysR family transcriptional regulator
PP_1580	52.25	-8.92	2.14E-03		hypothetical protein PP_1580
PP_3129	729.24	-4.60	2.15E-03	K01784	UDP-glucose 4-epimerase
PP_2263	102.61	-11.51	2.18E-03	K17323	binding-protein-dependent transport system inner membrane protein
PP_2350	41.48	-2.58	2.19E-03	K23773	LysR family transcriptional regulator
PP_2562	13.15	-3.85	2.20E-03		hypothetical protein PP_2562
PP_2261	17.33	-53.12	2.21E-03	K17325	sugar ABC transporter ATP-binding protein
PP_1548	337.97	-7.07	2.21E-03		hypothetical protein PP_1548
PP_3420	369.61	-4.12	2.21E-03		sensor histidine kinase
PP_2482	26.82	-3.43	2.23E-03	K03639	molybdenum cofactor biosynthesis protein A
PP_2480	48.53	-2.88	2.23E-03		xanthine dehydrogenase accessory factor
PP_4037	957.20	-4.90	2.23E-03	K17722	oxidoreductase
PP_2577	195.22	-3.69	2.23E-03	K06192	hypothetical protein PP_2577
PP_1132	100.09	-2.65	2.23E-03	K03313	pH-dependent sodium/proton antiporter
PP_1493	441.00	-3.26	2.25E-03	K13491	chemotaxis-specific methylesterase
PP_2422	57.74	-7.42	2.27E-03		alkylhydroperoxidase
PP_3005	46.10	-3.01	2.27E-03		hypothetical protein PP_3005
PP_2575	99.41	-3.86	2.27E-03		hypothetical protein PP_2575
PP_3565	54.17	-13.07	2.27E-03		amino acid transporter LysE
PP_2112	339.03	-3.69	2.28E-03	K01681	aconitate hydratase
PP_4657	1591.20	-2.23	2.28E-03	K07054	metallopeptidase. zinc binding
PP_2851	43.67	-4.66	2.29E-03		hypothetical protein PP_2851
PP_1811	70.30	-6.21	2.30E-03	K01791	UDP-N-acetylglucosamine 2-epimerase
PP_1937	603.89	-2.04	2.30E-03		helicase
PP_1956	280.16	-15.46	2.31E-03		hypothetical protein PP_1956
PP_3015	7.01	-6.00	2.33E-03		medium chain acyl-CoA ligase
PP_5242	517.58	-2.15	2.33E-03		sensor histidine kinase/GAF domain-containing protein
PP_2996	141.70	-2.45	2.34E-03		hypothetical protein PP_2996
PP_3956	99.34	-3.62	2.35E-03		hypothetical protein PP_3956
PP_3444	15.15	-9.11	2.36E-03	K04750	glyoxalase/bleomycin resistance protein/dioxygenase
PP_5323	279.06	-11.57	2.39E-03		M24/M37 family peptidase
PP_2349	10.79	-9.08	2.41E-03	K07168	hypothetical protein PP_2349
PP_1035	45.47	-2.48	2.44E-03		cumB protein
PP_1364	683.46	-4.10	2.44E-03		type IV pilus assembly PilZ
PP_1416	101.66	-2.42	2.44E-03	K07793	tricarboxylate transport protein TctA
PP_3682	44.60	-2.31	2.44E-03		hypothetical protein PP_3682
PP_0907	136.73	-3.68	2.44E-03	K03585	RND efflux membrane fusion protein-like protein

Gene ID	baseMean (normalized)	log2 Fold Change	p-value	KO term	Description
PP_2647	107.93	-3.10	2.46E-03		major facilitator family transporter
PP_2007	112.93	-6.68	2.46E-03		P-47-like protein
PP_0277	21.65	-2.21	2.46E-03		hypothetical protein PP_0277
PP_1481	433.25	-3.53	2.49E-03	K00137	gamma-aminobutyraldehyde dehydrogenase
PP_3427	59.92	-10.59	2.52E-03	K18300	NodT family RND efflux system outer membrane lipoprotein
PP_1561	35.72	-9.54	2.54E-03	K07451	phage holin
PP_0683	82.33	-4.31	2.55E-03		hypothetical protein PP_0683
PP_3249	134.36	-2.91	2.58E-03		aldo/keto reductase
PP_2926	37.36	-5.27	2.61E-03	K00012	UDP-glucose dehydrogenase
PP_4526	61.33	-2.34	2.61E-03		hypothetical protein PP_4526
PP_0570	1688.28	-3.03	2.61E-03	K06894	alpha-2-macroglobulin
PP_1547	85.23	-12.29	2.62E-03		hypothetical protein PP_1547
PP_1917	66.89	-2.77	2.62E-03	K02619	4-amino-4-deoxychorismate lyase
PP_1545	26.97	-26.18	2.63E-03		hypothetical protein PP_1545
PP_3104	432.21	-3.05	2.63E-03		hypothetical protein PP_3104
PP_1254	180.42	-2.31	2.65E-03		xenobiotic reductase A
PP_2572	155.08	-6.42	2.66E-03		hypothetical protein PP_2572
PP_1758	502.21	-2.76	2.68E-03	K07146	hypothetical protein PP_1758
PP_1492	784.19	-2.39	2.70E-03	K13490	chemotaxis protein CheA
PP_1849	118.81	-5.80	2.73E-03		hypothetical protein PP_1849
PP_1841	454.14	-4.47	2.73E-03		cytochrome c family protein
PP_1552	18.69	-11.79	2.74E-03	K02315	IstB domain-containing protein ATP-binding protein
PP_3581	137.43	-2.46	2.74E-03	K21023	diguanylate cyclase
PP_4060	266.63	-4.07	2.78E-03	K16147	alpha-amylase
PP_2392	91.62	-2.52	2.79E-03		major facilitator family transporter
PP_1839	50.37	-3.01	2.81E-03		hypothetical protein PP_1839
PP_3108	4943.35	-2.08	2.82E-03		rhs-like protein
PP_4296	59.03	-2.46	2.83E-03		hypothetical protein PP_4296
PP_3401	49.45	-2.75	2.84E-03		hypothetical protein PP_3401
PP_2243	30.39	-2.78	2.87E-03		phosphoglycerate mutase
PP_1564	13.05	-7.20	2.88E-03		hypothetical protein PP_1564
PP_1660	15.28	-16.48	2.89E-03		hypothetical protein PP_1660
PP_3133	793.06	-4.03	2.89E-03		GMC oxidoreductase
PP_3127	1038.96	-2.84	2.89E-03	K25590	lipopolysaccharide biosynthesis protein
PP_2569	209.30	-9.95	2.90E-03		major facilitator superfamily transporter
PP_1947	57.44	-432.74	2.90E-03		hypothetical protein PP_1947
PP_1946	399.14	-171.66	2.91E-03		short chain dehydrogenase/reductase oxidoreductase
PP_2578	50.50	-4.91	2.92E-03	K09857	hypothetical protein PP_2578
PP_1542	333.88	-3.34	2.92E-03		hypothetical protein PP_1542
PP_3602	157.75	-2.29	2.93E-03	K13877	ketoglutarate semialdehyde dehydrogenase
PP_1598	1123.92	-2.34	2.96E-03	K11749	zinc metalloprotease
PP_3913	48.43	-2.49	2.96E-03		hypothetical protein PP_3913
PP_2374	91.34	-2.62	2.97E-03		hypothetical protein PP_2374
PP_4689	89.63	-4.13	2.98E-03	K25132	hypothetical protein PP_4689
PP_1661	167.03	-8.86	3.01E-03	K19813	dehydrogenase subunit
PP_3435	222.57	-2.36	3.01E-03		diguanylate phosphodiesterase
PP_1583	9.13	-12.34	3.04E-03		structural protein P5
PP_5273	146.49	-38.15	3.04E-03	K09471	oxidoreductase
PP_3414	499.41	-3.69	3.07E-03	K03406	methyl-accepting chemotaxis transducer/sensory box protein
PP_4959	2479.19	-3.56	3.08E-03		response regulator receiver modulated diguanylate cyclase/phosphodiesterase with PAS/PAC sensor(s)
PP_3076	106.44	-2.83	3.11E-03	K13895	binding-protein-dependent transport system inner membrane protein
PP_2793	12.02	-41.61	3.17E-03	K00249	acyl-CoA dehydrogenase
PP_2576	93.38	-5.88	3.17E-03		hypothetical protein PP_2575
PP_0580	60.77	-3.48	3.18E-03		acyl dehydratase MaoC
PP_1073	223.73	-7.97	3.19E-03	K00111	glycerol-3-phosphate dehydrogenase
PP_2022	95.11	-3.85	3.22E-03		hypothetical protein PP_2022
PP_3579	78.19	-2.91	3.23E-03		hypothetical protein PP_3579
PP_3734	64.17	-3.62	3.24E-03	K02004	hypothetical protein PP_3734
PP_1102	44.91	-2.16	3.24E-03		hypothetical protein PP_1102
PP_1582	125.00	-5.51	3.26E-03		hypothetical protein PP_1582
PP_1510	203.88	-2.07	3.27E-03		carboxyvinyl-carboxyphosphonate phosphorylmutase
PP_1563	257.30	-6.85	3.28E-03		phage terminase. large subunit
PP_1683	85.81	-5.82	3.29E-03		MarR family transcriptional regulator
PP_2827	2124.22	-2.22	3.31E-03		zinc-containing alcohol dehydrogenase
PP_2526	286.93	-3.23	3.32E-03		hypothetical protein PP_2526
PP_5092	122.31	-5.44	3.33E-03		NLP/P60 protein
PP_3143	90.11	-2.13	3.34E-03	K03566	LysR family transcriptional regulator
PP_2511	2066.67	-2.63	3.39E-03		hypothetical protein PP_2511
PP_2362	269.29	-4.82	3.39E-03	K07347	fimbrial biogenesis outer membrane usher protein
PP_1368	109.84	-2.82	3.42E-03		hypothetical protein PP_1368
PP_2486	766.10	-3.09	3.43E-03	K10680	Oye family NADH-dependent flavin oxidoreductase
PP_0104	455.41	-6.19	3.46E-03	K02274	cytochrome c oxidase subunit I
PP_1762	1492.87	-2.69	3.52E-03		hypothetical protein PP_1762
PP_3569	372.64	-4.46	3.55E-03	K05358	quinate dehydrogenase
PP_2091	23.27	-2.84	3.56E-03		serine/threonine-protein kinase
PP_1540	57.29	-6.86	3.58E-03		hypothetical protein PP_1540
PP_5192	1380.66	-2.01	3.59E-03	K00281	glycine dehydrogenase
PP_3107	39.63	-3.37	3.60E-03		hypothetical protein PP_3107
PP_1537	26.06	-5.53	3.60E-03		hypothetical protein PP_1537
PP_1659	55.72	-14.12	3.62E-03		hypothetical protein PP_1659
PP_3635	275.30	-2.52	3.62E-03	K02050	binding-protein-dependent transport system inner membrane protein
PP_3106	1123.50	-2.39	3.66E-03	K11904	hypothetical protein PP_3106
PP_2487	739.45	-2.40	3.67E-03		aldehyde dehydrogenase
PP_1034	97.82	-2.31	3.71E-03		multicopper oxidase
PP_3560	54.71	-2.75	3.71E-03		LysR family transcriptional regulator
PP_0106	247.78	-6.77	3.72E-03	K02276	cytochrome c oxidase subunit III
PP_0107	181.83	-8.35	3.75E-03	K14998	hypothetical protein PP_0107
PP_5225	419.43	-2.62	3.75E-03	K06202	frataxin-like protein
PP_2462	310.54	-2.01	3.77E-03		hypothetical protein PP_2462
PP_1579	210.62	-10.41	3.78E-03		hypothetical protein PP_1579
PP_2117	184.82	-2.21	3.82E-03	K03473	erythronate-4-phosphate dehydrogenase

Gene ID	baseMean (normalized)	log2 Fold Change	p-value	KO term	Description
PP_3081	226.53	-4.41	3.84E-03		PEBP family protein
PP_4257	127.70	-2.48	3.85E-03	K00407	cbb3-type cytochrome oxidase subunit
PP_4521	1738.68	-2.85	3.85E-03	K03776	aerotaxis receptor
PP_1565	155.29	-6.95	3.86E-03		HK97 family phage portal protein
PP_2841	98.74	-2.90	3.93E-03		phage integrase site specific recombinase
PP_3450	52.89	-2.60	3.98E-03	K11739	hypothetical protein PP_3450
PP_2387	704.31	-2.13	3.98E-03		hypothetical protein PP_2387
PP_3020	217.86	-2.27	3.99E-03	K07313	serine/threonine protein phosphatase
PP_2260	23.39	-49.25	3.99E-03	K17324	sugar ABC transporter ATP-binding protein
PP_3316	116.94	-2.15	3.99E-03	K03696	chaperone-associated ATPase
PP_1995	1083.98	-3.16	4.01E-03	K01817	N-(5\-phosphoribosyl)anthranilate isomerase
PP_3070	67.29	-2.69	4.02E-03	K03769	PpiC-type peptidyl-prolyl cis-trans isomerase
PP_3246	37.28	-3.12	4.03E-03	K15746	fatty acid hydroxylase
PP_4036	3333.59	-2.93	4.04E-03	K03457	NCS1 nucleoside transporter
PP_1999	186.98	-2.57	4.05E-03	K03558	colicin V production protein
PP_2006	246.05	-6.80	4.08E-03		hypothetical protein PP_2006
PP_2860	90.73	-3.40	4.10E-03	K03811	nicotinamide mononucleotide transporter
PP_1945	275.24	-82.59	4.11E-03	K01491	5.10-methylene-tetrahydrofolate dehydrogenase
PP_1556	13.44	-4.67	4.11E-03		hypothetical protein PP_1556
PP_1535	18.17	-14.27	4.12E-03		methyltransferase
PP_1541	85.42	-13.62	4.12E-03	K00558	methyltransferase
PP_4042	337.72	-2.24	4.14E-03	K00036	glucose-6-phosphate 1-dehydrogenase
PP_5420	493.35	-3.78	4.15E-03	K02116	F0F1 ATP synthase subunit I
PP_4138	1103.32	-3.21	4.15E-03	K19784	NADPH-dependent FMN reductase
PP_2713	330.05	-2.24	4.17E-03	K07666	DNA-binding response regulator
PP_0108	29.23	-13.33	4.19E-03		hypothetical protein PP_0108
PP_1577	262.93	-8.66	4.22E-03		lambda family phage tail tape measure protein
PP_2970	77.26	-2.31	4.25E-03		hypothetical protein PP_2970
PP_1578	88.81	-6.83	4.30E-03		hypothetical protein PP_1578
PP_3413	205.77	-2.36	4.30E-03	K07679	Hpt sensor hybrid histidine kinase
PP_1760	210.51	-4.12	4.31E-03	K06148	ABC transporter ATP-binding protein
PP_2794	13.32	-18.85	4.31E-03		short chain dehydrogenase/reductase oxidoreductase
PP_0718	296.41	-2.88	4.32E-03		sulfate transporter
PP_2291	52.97	-4.86	4.35E-03		hypothetical protein PP_2291
PP_2560	98.65	-4.31	4.36E-03	K12536	type I secretion system ATPase
PP_1225	68.65	-4.10	4.36E-03	K10026	radical SAM domain-containing protein
PP_1538	49.24	-11.93	4.36E-03		hypothetical protein PP_1538
PP_3497	684.08	-2.01	4.37E-03	K24847	U32 family peptidase
PP_5086	87.36	-3.60	4.37E-03		nuclease
PP_1490	265.94	-3.10	4.37E-03	K13486	methyltransferase. CheR-like
PP_3136	207.74	-4.18	4.38E-03	K00640	serine O-acetyltransferase
PP_4576	161.49	-3.09	4.38E-03		allophanate hydrolase subunit 1
PP_1957	293.15	-5.31	4.40E-03		Pdr/VanB family oxidoreductase
PP_4010	5636.49	-2.05	4.40E-03	K03704	cold-shock protein CspD
PP_2237	57.63	-2.27	4.44E-03		hypothetical protein PP_2237
PP_1633	246.44	-2.49	4.45E-03		hypothetical protein PP_1633
PP_ICEclcD	13377.97	-2.43	4.45E-03	K02116	PP_aacC1
PP_4647	467.59	-3.16	4.46E-03		LuxR family transcriptional regulator
PP_0679	20601.71	-4.82	4.48E-03		hypothetical protein PP_0679
PP_1536	17.43	-11.13	4.48E-03		hypothetical protein PP_1536
PP_4508	1869.15	-2.14	4.49E-03		AraC family transcriptional regulator
PP_5190	41.84	-2.60	4.49E-03	K02454	type II secretion system protein E
PP_1412	454.86	-3.88	4.51E-03		enoyl-CoA hydratase
PP_1226	156.75	-2.14	4.51E-03	K06920	exoenzyme S synthesis protein B
PP_1759	131.10	-4.02	4.53E-03	K07396	DSBA oxidoreductase
PP_2025	65.59	-2.02	4.54E-03	K03547	nuclease SbcCD subunit D
PP_2646	21.85	-2.93	4.54E-03		hypothetical protein PP_2646
PP_4051	566.41	-2.79	4.55E-03	K01236	malto-oligosyltrehalose trehalohydrolase
PP_3016	49.16	-3.41	4.56E-03		lipopolysaccharide core biosynthesis protein
PP_3313	273.61	-2.19	4.56E-03		heat shock protein
PP_2944	356.00	-3.05	4.59E-03		sensor histidine kinase
PP_2485	169.35	-2.16	4.60E-03		hypothetical protein PP_2485
PP_3415	315.57	-2.44	4.61E-03	K06145	LacI family transcriptional regulator
PP_2769	235.95	-5.89	4.62E-03	K01998	branched-chain amino acid ABC transporter permease
PP_1134	429.55	-2.62	4.63E-03	K07137	FAD dependent oxidoreductase
PP_2196	96.50	-2.45	4.68E-03	K01480	agmatinase
PP_3580	296.81	-4.23	4.71E-03		hypothetical protein PP_3580
PP_4256	683.97	-2.12	4.71E-03	K00405	cbb3-type cytochrome c oxidase subunit II
PP_1771	2019.63	-2.88	4.71E-03	K00945	cytidylate kinase
PP_1825	133.59	-3.43	4.72E-03		hypothetical protein PP_1825
PP_2255	145.81	-2.59	4.72E-03	K11103	C4-dicarboxylate transporter DctA
PP_3418	67.23	-3.43	4.72E-03		hypothetical protein PP_3418
PP_4604	131.64	-8.33	4.73E-03		hypothetical protein PP_4604
PP_0806	1259.22	-2.70	4.73E-03	K20276	surface adhesion protein
PP_3030	205.91	-2.67	4.76E-03		hypothetical protein PP_3030
PP_2347	81.81	-2.39	4.82E-03		winged helix family two component transcriptional regulator
PP_3396	23.98	-2.54	4.86E-03		hypothetical protein PP_3396
PP_2011	253.59	-3.03	4.86E-03	K09977	hypothetical protein PP_2011
PP_1801	400.02	-2.18	4.87E-03		glycosyl transferase WbpY
PP_1567	332.76	-3.94	4.87E-03		HK97 family phage major capsid protein
PP_3449	47.55	-4.26	4.88E-03		hypothetical protein PP_3449
PP_2309	202.98	-2.67	4.91E-03		hypothetical protein PP_2309
PP_2579	99.24	-4.53	4.97E-03	K19353	hypothetical protein PP_2579
PP_1755	453.04	-2.21	4.98E-03	K01679	fumarate hydratase
PP_0922	298.28	-4.66	4.99E-03	K08682	hypothetical protein PP_0922
PP_2360	246.34	-4.01	5.00E-03		spore coat U domain-containing protein
PP_1396	60.66	-4.34	5.02E-03		antibiotic biosynthesis monooxygenase
PP_1560	14.69	-5.74	5.04E-03		hypothetical protein PP_1560
PP_1562	47.89	-10.01	5.05E-03		phage terminase small subunit
PP_1918	210.32	-2.07	5.06E-03	K07082	aminodeoxychorismate lyase
PP_1572	65.01	-3.97	5.11E-03		hypothetical protein PP_1572
PP_2598	81.79	-5.24	5.12E-03		hypothetical protein PP_2598
PP_4559	210.18	-2.27	5.13E-03	K01462	peptide deformylase

Gene ID	baseMean (normalized)	log2 Fold Change	p- value	KO term	Description
PP_2338	325.05	-2.31	5.13E-03	K01720	2-methylcitrate dehydratase
PP_5222	356.29	-2.24	5.13E-03	K05851	adenylate cyclase
PP_0717	151.25	-2.22	5.15E-03		hypothetical protein PP_0717
PP_1687	71.66	-2.97	5.18E-03		hypothetical protein PP_1687
PP_2792	7.73	-11.53	5.21E-03		hypothetical protein PP_2792
PP_1534	51.22	-5.34	5.23E-03		hypothetical protein PP_1534
PP_1554	7.42	-5.57	5.24E-03		hypothetical protein PP_1554
PP_2233	107.28	-2.59	5.27E-03		isochorismatase superfamily hydrolase
PP_3142	1336.97	-2.93	5.28E-03		sugar transferase
PP_2162	34.83	-4.59	5.32E-03		hypothetical protein PP_2162
PP_3157	259.45	-3.15	5.33E-03		inositol monophosphatase
PP_2130	927.29	-2.21	5.36E-03	K08309	lytic transglycosylase
PP_2332	104.28	-3.27	5.37E-03		hypothetical protein PP_2332
PP_ICEint	66.57	-2.60	5.37E-03		PP_aacC1
PP_2707	24.98	-3.33	5.42E-03	K01142	exodeoxyribonuclease III
PP_3075	153.88	-3.02	5.44E-03		Fis family transcriptional regulator
PP_0778	132.73	-2.71	5.47E-03		glycoside hydrolase family protein
PP_3517	73.39	-5.08	5.47E-03		hypothetical protein PP_3517
PP_3105	614.64	-3.14	5.49E-03		hypothetical protein PP_3105
PP_1559	20.13	-6.34	5.49E-03		phage holin
PP_1686	2498.28	-2.22	5.49E-03	K00432	glutathione peroxidase
PP_3636	122.68	-2.78	5.57E-03	K02051	sulfonate ABC transporter substrate-binding protein
PP_2118	110.30	-2.93	5.58E-03		hypothetical protein PP_2118
PP_2307	387.38	-4.09	5.59E-03		CHAD domain-containing protein
PP_1429	951.21	-2.08	5.61E-03	K03598	sigma E regulatory protein MucB/RseB
PP_3518	178.73	-3.43	5.66E-03		hypothetical protein PP_3518
PP_3006	27.13	-2.51	5.67E-03	K03088	RNA polymerase sigma factor
PP_4270	282.32	-2.84	5.67E-03	K02030	hypothetical protein PP_4270
PP_1951	141.65	-143.54	5.71E-03		short chain dehydrogenase/reductase oxidoreductase
PP_5307	43.68	-2.55	5.73E-03	K03559	biopolymer transport protein ExbD
PP_4118	103.61	-3.36	5.76E-03		hypothetical protein PP_4118
PP_2051	64.90	-2.19	5.77E-03	K00626	acetyl-CoA acetyltransferase
PP_1546	21.75	-18.23	5.87E-03		hypothetical protein PP_1546
PP_4207	154.61	-3.85	5.88E-03		hypothetical protein PP_4207
PP_1574	43.64	-5.79	5.91E-03		hypothetical protein PP_1574
PP_3940	124.28	-2.04	5.92E-03		major facilitator family transporter
PP_2421	120.12	-2.64	6.00E-03		acetyltransferase
PP_4061	182.73	-4.00	6.01E-03		hypothetical protein PP_4061
PP_0386	1130.14	-2.08	6.04E-03		sensory box protein
PP_2661	13.35	-2.54	6.06E-03		hypothetical protein PP_2661
PP_2660	69.03	-2.25	6.09E-03		hypothetical protein PP_2660
PP_2872	379.04	-2.16	6.14E-03	K15268	hypothetical protein PP_2872
PP_0716	105.88	-3.69	6.15E-03		secretion protein HlyD family protein
PP_1919	348.55	-2.15	6.15E-03	K07082	aminodeoxychorismate lyase
PP_1596	370.74	-2.49	6.16E-03	K00981	phosphatidate cytidylyltransferase
PP_0804	156.16	-2.05	6.17E-03	K12541	protein secretion ABC efflux system. permease and ATP-binding protein
PP_0023	186.42	-2.77	6.21E-03		hypothetical protein PP_0023
PP_1640	716.18	-11.40	6.21E-03		hypothetical protein PP_1640
PP_3549	249.58	-2.33	6.23E-03	K03543	secretion protein HlyD family protein
PP_1488	812.88	-2.18	6.24E-03	K13487	methyl-accepting chemotaxis sensory transducer
PP_1570	75.67	-2.91	6.24E-03		head-tail adaptor
PP_4799	507.56	-2.62	6.32E-03	K01297	muramoyltetrapeptide carboxypeptidase
PP_3132	1203.01	-4.00	6.34E-03		polysaccharide biosynthesis protein
PP_2361	254.45	-4.26	6.35E-03	K07346	type 1 pili usher pathway chaperone CsuC
PP_0572	155.69	-2.63	6.37E-03	K05367	penicillin-binding protein 1C
PP_2966	34.39	-2.50	6.38E-03		hypothetical protein PP_2966
PP_4469	830.97	-2.57	6.41E-03	K05774	phosphonate metabolism protein/1.5-bisphosphokinase PhnN
PP_2877	140.74	-2.93	6.43E-03	K14347	bile acid/Na+ symporter family protein
PP_2721	6.91	-3.13	6.45E-03		hypothetical protein PP_2721
PP_1550	27.18	-3.61	6.45E-03		Cro/CI family transcriptional regulator
PP_3432	214.20	-3.16	6.46E-03		hypothetical protein PP_3432
PP_5175	306.42	-2.51	6.54E-03	K21136	RND family efflux transporter MFP subunit
PP_2373	503.31	-5.71	6.57E-03		hypothetical protein PP_2373
PP_0989	152.70	-2.23	6.62E-03	K02437	glycine cleavage system protein H
PP_3751	66.83	-2.13	6.62E-03	K03449	major facilitator superfamily transporter
PP_0365	264.02	-2.63	6.62E-03	K02169	biotin biosynthesis protein BioC
PP_3629	237.96	-4.09	6.67E-03		3-demethylubiquinone-9 3-methyltransferase
PP_0798	89.83	-2.42	6.68E-03		diguanylate cyclase
PP_2828	33.04	-2.13	6.68E-03		hypothetical protein PP_2828
PP_1597	538.12	-2.33	6.69E-03	K00099	1-deoxy-D-xylulose 5-phosphate reductoisomerase
PP_1369	255.49	-3.20	6.69E-03	K06986	hypothetical protein PP_1369
PP_2289	14.05	-3.12	6.73E-03		hypothetical protein PP_2289
PP_1239	315.21	-2.21	6.74E-03		beta-lactamase domain-containing protein
PP_3131	766.89	-3.59	6.76E-03		hypothetical protein PP_3131
PP_1916	601.72	-3.28	6.83E-03	K09458	3-oxoacyl-ACP synthase
PP_3512	78.69	-3.40	6.83E-03		transmembrane pair domain-containing protein
PP_4314	231.34	-2.72	6.85E-03		hypothetical protein PP_4314
PP_3609	226.44	-2.09	6.87E-03	K09936	hypothetical protein PP_3609
PP_3126	1776.52	-2.80	6.89E-03	K01991	polysaccharide export protein
PP_2943	199.26	-3.48	6.95E-03	K00428	cytochrome c551 peroxidase
PP_2150	355.29	-2.44	6.95E-03	K03734	ApbE family lipoprotein
PP_1121	1366.83	-4.25	6.99E-03		OmpA/MotB domain-containing protein
PP_3463	67.10	-3.90	7.00E-03	K00146	aldehyde dehydrogenase
PP_1571	128.05	-5.37	7.00E-03		hypothetical protein PP_1571
PP_ICE10938	13466.46	-2.01	7.04E-03	K02116	PP_aacC1
PP_3624	310.78	-2.71	7.07E-03	K02030	hypothetical protein PP_3624
PP_4694	115.22	-2.03	7.14E-03	K01894	glutamyl-Q tRNA(Asp) synthetase
PP_2520	0.55	-6.49	7.19E-03		hypothetical protein PP_2520
PP_2095	327.22	-2.48	7.21E-03	K00254	dihydroorotate dehydrogenase 2
PP_4211	28.77	-2.33	7.21E-03		RND efflux transporter
PP_2945	423.28	-3.00	7.25E-03	K02482	sensor histidine kinase/response regulator
PP_t25	2.73	-4.79	7.25E-03		PP_aacC1
PP_4254	57.28	-5.03	7.29E-03		hypothetical protein PP_4254
PP_3250	100.94	-2.09	7.29E-03	K03301	major facilitator superfamily transporter
PP_0871	51.92	-2.60	7.30E-03	K05846	binding-protein-dependent transport system inner membrane protein
PP_0086	297.26	-3.03	7.33E-03	K09165	hypothetical protein PP_0086
PP_1727	124.18	-2.13	7.34E-03		GntR family transcriptional regulator
PP_1609	64.91	-4.18	7.43E-03		hypothetical protein PP_1609
PP_1800	597.38	-2.33	7.44E-03	K22252	oxidoreductase Rmd
PP_1029	52.29	-2.06	7.48E-03	K07090	hypothetical protein PP_1029
PP_1871	358.16	-2.02	7.52E-03	K03799	heat shock protein HtpX
PP_3421	614.16	-3.83	7.52E-03		sensor histidine kinase
PP_2477	20.22	-2.07	7.60E-03	K07302	isoquinoline 1-oxidoreductase subunit alpha
PP_1420	143.66	-2.13	7.65E-03	K07774	winged helix family two component transcriptional regulator
PP_0103	493.75	-7.17	7.66E-03	K02275	cytochrome c oxidase subunit II
PP_2048	36.52	-2.24	7.68E-03		acyl-CoA dehydrogenase
PP_0216	159.46	-2.30	7.69E-03		PAS/PAC sensor-containing diguanylate cyclase
PP_4793	1166.18	-5.03	7.70E-03		hypothetical protein PP_4793
PP_3944	30.20	-3.19	7.72E-03	K14974	salicylate hydroxylase
PP_2252	53.20	-3.30	7.73E-03		hypothetical protein PP_2252
PP_3242	525.12	-3.33	7.74E-03		diguanylate cyclase
PP_2476	206.45	-2.20	7.76E-03	K23256	zinc-containing alcohol dehydrogenase
PP_2896	25.46	-2.22	7.79E-03		hypothetical protein PP_2896
PP_4734	620.29	-2.12	7.82E-03	K05799	GntR family transcriptional regulator
PP_1184	907.35	-2.02	7.86E-03		dienelactone hydrolase
PP_0210	12.87	-2.26	7.89E-03		phycobiliprotein
PP_3567	184.95	-2.06	7.90E-03		LysR family transcriptional regulator
PP_2326	274.43	-5.70	7.91E-03		universal stress protein
PP_1539	131.18	-4.22	7.92E-03		hypothetical protein PP_1539
PP_4738	18.23	-3.19	7.96E-03		hypothetical protein PP_4738
PP_0433	762.51	-2.34	7.98E-03	K15724	iron-sulfur cluster insertion protein ErpA
PP_1387	144.26	-2.97	8.00E-03	K03577	TetR family transcriptional regulator
PP_2241	105.87	-3.96	8.03E-03		major facilitator family transporter
PP_1568	45.34	-3.06	8.05E-03		hypothetical protein PP_1568
PP_0714	356.89	-3.16	8.09E-03		permease
PP_2064	13.97	-2.58	8.10E-03	K18306	RND family efflux transporter MFP subunit
PP_3134	1748.04	-2.53	8.12E-03		transferase hexapeptide repeat containing protein
PP_3412	155.36	-2.28	8.13E-03		LuxR family transcriptional regulator
PP_2726	10.56	-2.62	8.14E-03		hypothetical protein PP_2726
PP_3941	40.69	-2.54	8.14E-03	K13995	isochorismatase superfamily hydrolase
PP_5386	55.90	-2.21	8.19E-03	K07798	CzcB family heavy metal RND efflux membrane fusion protein
PP_1467	476.89	-2.32	8.22E-03		sodium/hydrogen exchanger
PP_2791	15.17	-55.43	8.30E-03		aminoglycoside phosphotransferase
PP_3156	974.84	-4.48	8.33E-03		universal stress protein
PP_2510	69.37	-2.99	8.39E-03		hypothetical protein PP_2510
PP_0921	141.42	-2.05	8.46E-03		ArsR family transcriptional regulator
PP_1346	744.14	-2.14	8.52E-03	K00620	bifunctional ornithine acetyltransferase/N-acetylglutamate synthase
PP_3145	1498.67	-2.28	8.65E-03		hypothetical protein PP_3145
PP_2405	76.54	-2.27	8.69E-03		redoxin domain-containing protein
PP_5218	62.60	-2.05	8.78E-03		DedA family protein
PP_2415	83.65	-2.59	8.82E-03		acetyltransferase
PP_2698	410.29	-2.68	8.89E-03	K00549	5-methyltetrahydropteroyltriglutamate/homocysteine S-methyltransferase
PP_4714	355.66	-2.28	8.89E-03	K09748	hypothetical protein PP_4714
PP_3735	58.85	-3.60	8.93E-03	K02003	ABC transporter ATP-binding protein
PP_1478	1016.23	-2.36	9.02E-03		NADH:flavin oxidoreductase
PP_2941	236.88	-3.03	9.02E-03		hypothetical protein PP_2941
PP_2251	49.01	-4.70	9.03E-03		transmembrane pair domain-containing protein
PP_2417	18.83	-2.54	9.07E-03	K02015	transport system permease
PP_3312	262.26	-2.36	9.12E-03	K13993	heat shock protein
PP_2942	879.76	-3.40	9.17E-03		response regulator
PP_0164	154.29	-2.11	9.19E-03		hypothetical protein PP_0164
PP_4711	79.11	-2.50	9.23E-03	K02834	ribosome-binding factor A
PP_3144	149.66	-4.60	9.25E-03	K01752	iron-sulfur-dependent L-serine dehydratase
PP_2493	8.32	-2.87	9.30E-03	K03630	RadC family protein
PP_4966	84.86	-5.73	9.33E-03		ArsR family transcriptional regulator
PP_4556	32.68	-2.89	9.35E-03		hypothetical protein PP_4556
PP_0536	223.23	-3.54	9.39E-03		hypothetical protein PP_0536
PP_4858	765.81	-2.38	9.39E-03		hypothetical protein PP_4858
PP_2728	82.13	-2.01	9.46E-03		hypothetical protein PP_2728
PP_2244	222.35	-2.09	9.53E-03	K07090	hypothetical protein PP_2244
PP_1566	77.47	-5.25	9.66E-03	K06904	head maturation protease
PP_3153	27.77	-2.14	9.69E-03		hypothetical protein PP_3153
PP_1418	147.33	-2.08	9.76E-03	K07795	tricarboxylate transport protein TctC
PP_2290	4.33	-3.96	9.77E-03		hypothetical protein PP_2290
PP_0560	329.15	-3.70	9.82E-03	K03786	3-dehydroquinate dehydratase
PP_1991	1.10	-7.56	9.82E-03		hypothetical protein PP_1991
PP_2596	22.88	-2.55	1.00E-02	K14698	ABC transporter ATP-binding protein/permease
PP_3756	27.03	-2.26	1.00E-02		TetR family transcriptional regulator
PP_4878	390.45	-2.65	1.01E-02	K02990	30S ribosomal protein S6
PP_0102	363.69	-2.75	1.02E-02		hypothetical protein PP_0102
PP_5210	290.17	-2.01	1.02E-02	K00344	zinc-containing alcohol dehydrogenase
PP_4145	182.15	-4.87	1.03E-02	K08307	lytic murein transglycosylase D
PP_1734	153.16	-2.06	1.03E-02	K03610	septum formation inhibitor
PP_3568	10.23	-2.49	1.04E-02	K11312	hypothetical protein PP_3568
PP_1573	80.31	-4.57	1.05E-02		major tail protein
PP_2026	140.70	-2.34	1.05E-02		hypothetical protein PP_2026
PP_1095	59.49	-2.76	1.05E-02	K03614	RnfABCDGE type electron transport complex subunit D
PP_2188	63.04	-2.38	1.06E-02	K06169	tRNA--hydroxylase
PP_4617	996.06	-3.39	1.06E-02	K00263	leucine dehydrogenase
PP_2389	1490.86	-2.61	1.07E-02	K09190	hypothetical protein PP_2389
PP_4557	112.79	-3.44	1.09E-02		hypothetical protein PP_4557
PP_2021	436.69	-2.49	1.10E-02		hypothetical protein PP_2021

Gene ID	baseMean (normalized)	log2 Fold Change	p- value	KO term	Description
PP_3004	151.91	-2.48	1.13E-02		hypothetical protein PP_3004
PP_1929	30.74	-2.34	1.13E-02	K03893	arsenite efflux transporter
PP_4973	931.80	-2.32	1.15E-02		hypothetical protein PP_4973
PP_3323	4.84	-2.26	1.15E-02		CobW/P47K family protein
PP_3550	25.61	-5.25	1.16E-02		MarR family transcriptional regulator
PP_ICE66625	0.68	-4.04	1.17E-02	K02116	PP_aacC1
PP_4901	99.47	-2.01	1.17E-02		hypothetical protein PP_4901
PP_2729	2086.48	-2.54	1.17E-02		hypothetical protein PP_2729
PP_1575	11.91	-2.56	1.18E-02		hypothetical protein PP_1575
PP_2062	194.59	-2.00	1.18E-02		hypothetical protein PP_2062
PP_3128	734.06	-2.79	1.19E-02	K00903	protein-tyrosine kinase
PP_3293	398.90	-2.50	1.19E-02		hypothetical protein PP_3293
PP_1533	2.27	-12.42	1.20E-02		excisionase
PP_2379	57.48	-3.32	1.20E-02	K07152	electron transport protein SCO1/SenC
PP_2268	2.18	-3.19	1.20E-02		phage endodeoxyribonuclease I
PP_3371	42.16	-2.03	1.24E-02		integral membrane sensor signal transduction histidine kinase
PP_3007	11.73	-3.32	1.24E-02		hypothetical protein PP_3007
PP_1641	286.38	-2.14	1.25E-02	K14058	C32 tRNA thiolase
PP_1244	232.95	-3.10	1.29E-02		hypothetical protein PP_1244
PP_1448	159.80	-2.17	1.30E-02		hypothetical protein PP_1448
PP_2559	78.72	-2.36	1.30E-02	K12537	HlyD family type I secretion membrane fusion protein
PP_3542	8.50	-2.51	1.32E-02		hypothetical protein PP_3542
PP_4688	51.57	-2.22	1.32E-02	K25133	transport system permease
PP_3233	109.42	-3.17	1.32E-02		Crp/Fnr family transcriptional regulator
PP_0351	19.92	-2.17	1.33E-02	K07165	FecR anti-FecI sigma factor
PP_0866	25.36	-2.64	1.33E-02	K07165	FecR anti-FecI sigma factor
PP_2979	13.18	-2.16	1.34E-02		hypothetical protein PP_2979
PP_1324	73.63	-2.02	1.34E-02	K07460	hypothetical protein PP_1324
PP_2377	76.63	-2.15	1.35E-02		acyltransferase
PP_1525	83.90	-2.13	1.36E-02	K01439	succinyl-diaminopimelate desuccinylase
PP_0962	4599.35	-2.38	1.38E-02	K07122	toluene-tolerance protein
PP_0713	430.31	-3.88	1.39E-02	K11747	potassium efflux system protein
PP_0175	57.18	-3.46	1.39E-02	K06075	MarR family transcriptional regulator
PP_3018	14.95	-2.56	1.40E-02		hypothetical protein PP_3018
PP_1655	825.77	-2.03	1.40E-02	K03215	23S rRNA 5-methyluridine methyltransferase
PP_0176	175.55	-2.33	1.40E-02		fusaric acid resistance protein
PP_0364	291.84	-2.10	1.42E-02	K02170	carboxylesterase
PP_2438	127.07	-2.09	1.43E-02	K05794	hypothetical protein PP_2438
PP_3531	149.38	-2.35	1.44E-02		lipid-binding START domain-containing protein
PP_0712	1253.94	-2.25	1.44E-02	K22468	hypothetical protein PP_0712
PP_3099	53.85	-2.78	1.45E-02	K11900	hypothetical protein PP_3099
PP_0110	196.74	-2.39	1.45E-02	K02257	protoheme IX farnesyltransferase
PP_0957	312.08	-2.21	1.45E-02	K06041	KpsF/GutQ family protein
PP_1458	415.00	-2.04	1.46E-02		metabolite-proton symporter
PP_0393	50.40	-2.04	1.47E-02	K00950	2-amino-4-hydroxy-6-hydroxymethyldihydropteridine pyrophosphokinase
PP_3054	4.52	-2.43	1.48E-02		pyocin R2_PP. baseplate/tail fiber protein
PP_3916	33.56	-3.40	1.48E-02		hypothetical protein PP_3916
PP_2103	365.98	-2.07	1.52E-02		hypothetical protein PP_2103
PP_1642	69.17	-2.18	1.52E-02		hypothetical protein PP_1642
PP_1456	43.72	-2.27	1.53E-02		hypothetical protein PP_1456
PP_1969	64.99	-2.10	1.54E-02	K03639	molybdenum cofactor biosynthesis protein A
PP_2158	20.39	-2.02	1.54E-02	K07665	two component heavy metal response transcriptional regulator
PP_2245	65.38	-2.03	1.55E-02		Cro/CI family transcriptional regulator
PP_2744	94.24	-2.09	1.56E-02	K00948	ribose-phosphate pyrophosphokinase
PP_1491	192.32	-2.12	1.61E-02	K13489	chemotaxis protein CheW
PP_1425	95.75	-2.35	1.62E-02	K19168	hypothetical protein PP_1425
PP_1925	69.71	-2.23	1.63E-02		monooxygenase
PP_2449	333.60	-2.20	1.64E-02		sporulation domain-containing protein
PP_4585	132.63	-2.19	1.65E-02		RNA methyltransferase
PP_4629	195.18	-2.05	1.65E-02		sensory box protein
PP_1507	45.97	-2.51	1.66E-02	K14742	hypothetical protein PP_1507
PP_2935	96.97	-2.01	1.66E-02		major facilitator superfamily transporter
PP_1837	368.22	-2.10	1.67E-02		hypothetical protein PP_1837
PP_2717	35.73	-2.14	1.70E-02	K03893	arsenite efflux transporter
PP_0755	93.39	-3.44	1.70E-02		hypothetical protein PP_0755
PP_4890	1431.30	-2.02	1.71E-02	K02502	ATP phosphoribosyltransferase
PP_2597	34.41	-2.27	1.72E-02		hypothetical protein PP_2597
PP_4363	1235.39	-3.28	1.72E-02	K20977	response regulator receiver protein
PP_2453	604.27	-5.63	1.72E-02	K05597	type II L-asparaginase
PP_2464	462.42	-2.30	1.72E-02		hypothetical protein PP_2464
PP_1553	18.23	-3.65	1.73E-02		hypothetical protein PP_1553
PP_4534	500.74	-3.24	1.73E-02		hypothetical protein PP_4534
PP_4710	2534.10	-2.09	1.74E-02	K02956	30S ribosomal protein S15
PP_2357	2149.34	-3.29	1.75E-02		spore coat U domain-containing protein
PP_2219	19.00	-11.34	1.76E-02		hypothetical protein PP_2219
PP_4117	48.72	-2.11	1.80E-02		hypothetical protein PP_4117
PP_2497	8.86	-2.17	1.81E-02		hypothetical protein PP_2497
PP_2699	514.15	-2.38	1.82E-02		hypothetical protein PP_2699
PP_0905	1003.40	-11.24	1.85E-02		hypothetical protein PP_0905
PP_1898	88.91	-2.59	1.88E-02	K03561	MotA/TolQ/ExbB proton channel
PP_3638	295.48	-3.01	1.93E-02		acyl-CoA dehydrogenase
PP_2337	649.09	-2.05	1.93E-02	K09788	hypothetical protein PP_2337
PP_2306	195.04	-5.35	1.95E-02		lipoprotein
PP_0703	10.83	-2.19	1.95E-02	K07165	FecR anti-FecI sigma factor
PP_1508	78.09	-2.13	2.01E-02		hypothetical protein PP_1508
PP_0352	5.19	-3.12	2.02E-02	K23514	RNA polymerase sigma factor
PP_1647	52.50	-2.24	2.02E-02	K03668	hypothetical protein PP_1647
PP_1833	49.23	-3.51	2.02E-02		hypothetical protein PP_1833
PP_2408	1.84	-2.91	2.04E-02	K15725	cobalt-zinc-cadmium resistance protein CzcC
PP_2435	49.97	-2.20	2.06E-02	K04487	cysteine desulfurase
PP_2878	13.41	-2.29	2.09E-02		hypothetical protein PP_2878
PP_4962	368.68	-2.34	2.10E-02		hypothetical protein PP_4962
PP_2855	20.75	-2.04	2.14E-02	K06992	hypothetical protein PP_2855
PP_0153	176.67	-2.01	2.14E-02		hypothetical protein PP_0153
PP_3637	89.50	-2.44	2.15E-02	K02049	sulfonate ABC transporter ATP-binding protein
PP_1555	186.94	-2.13	2.19E-02		phage integrase
PP_4735	691.82	-4.86	2.24E-02	K03303	L-lactate transport
PP_0111	580.17	-2.20	2.27E-02	K07152	electron transport protein SCO1/SenC
PP_3100	29.52	-4.09	2.31E-02	K11901	hypothetical protein PP_3100
PP_0258	3846.96	-2.20	2.31E-02		LysM domain/BON superfamily protein
PP_ICE81655	4.90	-2.85	2.35E-02	K02116	PP_aacC1
PP_2706	12.12	-2.76	2.38E-02		hypothetical protein PP_2706
PP_3442	2.41	-3.15	2.44E-02		hypothetical protein PP_3442
PP_4624	323.99	-2.55	2.50E-02		alpha/beta hydrolase
PP_2097	1788.72	-2.30	2.58E-02		PAS/PAC sensor-containing diguanylate cyclase
PP_3065	3.55	-3.15	2.63E-02	K06905	pyocin R2_PP. tail formation
PP_3631	25.80	-2.20	2.70E-02	K07184	hypothetical protein PP_3631
PP_1151	38.73	-2.16	2.71E-02		hypothetical protein PP_1151
PP_1840	633.01	-2.75	2.72E-02		hypothetical protein PP_1840
PP_0838	361.52	-3.11	2.73E-02	K01092	inositol-phosphate phosphatase
PP_1051	0.90	-3.54	2.80E-02	K02458	type II secretion system protein I/J
PP_1199	101.46	-2.04	2.82E-02		hypothetical protein PP_1199
PP_4724	367.47	-2.05	2.85E-02	K01956	carbamoyl phosphate synthase small subunit
PP_0357	1145.26	-3.13	2.92E-02		ACT domain-containing protein
PP_1527	109.65	-2.31	2.93E-02	K22132	UBA/THIF-type NAD/FAD binding protein
PP_0981	38.21	-3.14	2.94E-02		hypothetical protein PP_0981
PP_2141	658.52	-2.39	2.97E-02		hypothetical protein PP_2141
PP_2675	14.57	-38.77	3.02E-02		cytochrome c-type protein
PP_t55	3.90	-4.08	3.02E-02		PP_aacC1
PP_3139	1678.51	-2.16	3.08E-02		glycoside hydrolase family protein
PP_3522	3.09	-2.06	3.08E-02		endoribonuclease L-PSP
PP_5372	124.72	-2.18	3.09E-02		aldehyde dehydrogenase
PP_1531	66.13	-2.26	3.14E-02	K00537	arsenate reductase
PP_t45	12.32	-2.76	3.24E-02		PP_aacC1
PP_2759	9.96	-2.89	3.28E-02	K10441	ribose ABC transporter ATP-binding protein
PP_2359	777.92	-2.42	3.34E-02		spore coat U domain-containing protein
PP_5191	109.65	-2.01	3.35E-02		hypothetical protein PP_5191
PP_2663	47.82	-36.95	3.40E-02		hypothetical protein PP_2663
PP_ICE89746	30.96	-2.58	3.46E-02	K02116	PP_aacC1
PP_2672	76.42	-9.99	3.50E-02		LuxR family transcriptional regulator
PP_2450	636.95	-2.17	3.52E-02		hypothetical protein PP_2450
PP_3296	1166.44	-2.06	3.55E-02	K07112	hypothetical protein PP_3296
PP_t54	33.91	-2.20	3.56E-02		PP_aacC1
PP_2677	7.05	-14.93	3.60E-02	K17226	hypothetical protein PP_2677
PP_2669	19.36	-12.31	3.61E-02		hypothetical protein PP_2669
PP_1695	1284.51	-2.79	3.62E-02		integral membrane sensor hybrid histidine kinase
PP_3480	5.48	-2.89	3.63E-02		hypothetical protein PP_3480
PP_3851	289.65	-2.25	3.70E-02		hypothetical protein PP_3851
PP_2676	27.55	-22.26	3.74E-02		hypothetical protein PP_2676
PP_2670	27.81	-24.19	3.75E-02		hypothetical protein PP_2670
PP_2671	96.76	-28.08	3.91E-02	K07675	integral membrane sensor signal transduction histidine kinase
PP_1624	3.40	-2.24	4.05E-02		group II intron-encoding maturase
PP_2927	336.18	-4.15	4.11E-02		hypothetical protein PP_2927
PP_t52	23.58	-3.92	4.20E-02		PP_aacC1
PP_2680	107.55	-13.19	4.26E-02	K00138	aldehyde dehydrogenase
PP_3095	57.93	-2.11	4.36E-02	K11907	chaperone-associated ATPase
PP_2351	2562.00	-4.92	4.39E-02	K01908	acetyl-CoA synthetase
PP_t53	46.48	-2.17	4.40E-02		PP_aacC1
PP_2200	130.26	-2.39	4.47E-02		hypothetical protein PP_2200
PP_1924	78.22	-2.11	4.48E-02		phosphinothricin N-acetyltransferase
PP_2679	66.71	-12.43	4.54E-02	K00114	quinoprotein ethanol dehydrogenase
PP_0608	39.44	-3.10	4.58E-02	K02671	hypothetical protein PP_0608
PP_2061	4.00	-2.66	4.59E-02		hypothetical protein PP_2061
PP_3655	79.05	-2.00	4.60E-02	K03457	cytosine/purines uracil thiamine allantoin permease
PP_2858	8.53	-2.13	4.61E-02		hypothetical protein PP_2858
PP_3614	6.25	-2.06	4.62E-02		hypothetical protein PP_3614
PP_2681	91.86	-12.12	4.63E-02	K06138	pyrroloquinoline quinone biosynthesis protein PqqD
PP_2573	39.61	-2.35	4.96E-02		glyoxalase/bleomycin resistance protein/dioxygenase

Higher on benzoate

Gene ID	baseMean (normalized)	log2 Fold Change	p- value	KO term	Description
PP_0616	69.19	5.99	1.06E-04	K01995	branched chain amino acid ABC transporter ATP-binding protein
PP_4866	846.30	11.09	2.51E-04	K01997	inner-membrane translocator
PP_4264	508.38	8.84	2.53E-04	K02495	coproporphyrinogen III oxidase
PP_0445	6332.16	4.71	2.54E-04	K02864	50S ribosomal protein L10
PP_2168	1002.15	2.54	3.73E-04	K00616	transaldolase B
PP_0294	1588.37	4.01	4.54E-04	K02000	glycine betaine/L-proline ABC transporter ATP-binding subunit
PP_1299	659.69	14.55	4.86E-04	K09971	polar amino acid ABC transporter inner membrane subunit
PP_5207	1323.15	4.22	5.17E-04	K13926	ABC transporter ATP-binding protein/permease
PP_4252	6.10	50.81	5.19E-04	K00407	cytochrome c oxidase. cbb3-type. CcoQ subunit
PP_4274	531.32	2.12	5.65E-04	K01972	NAD-dependent DNA ligase LigA
PP_0316	295.67	7.98	6.08E-04	K21832	oxidoreductase FAD-binding domain-containing protein
PP_0859	1293.33	3.11	6.09E-04	K13566	nitrilase/cyanide hydratase and apolipoprotein N-acyltransferase
PP_4322	1641.91	2.23	6.18E-04	K02198	cytochrome C biogenesis protein CcmF
PP_3146	126.88	6.03	6.19E-04	K09471	oxidoreductase
PP_0282	4627.93	4.40	6.19E-04	K23055	amino acid ABC transporter substrate-binding protein
PP_5389	2592.81	4.68	6.84E-04		hypothetical protein PP_5389
PP_5348	136.59	2.74	7.35E-04	K21711	LysR family transcriptional regulator
PP_0310	149.38	10.26	8.08E-04	K21833	FMN-binding oxidoreductase
PP_5279	345.93	3.15	8.16E-04	K06995	hypothetical protein PP_5279

Gene ID	baseMean (normalized)	log2 Fold Change	p- value	KO term	Description
PP_0182	654.46	4.61	8.53E-04		hypothetical protein PP_0182
PP_4299	165.65	2.37	8.56E-04	K00042	2-hydroxy-3-oxopropionate reductase
PP_4594	347.41	2.06	8.70E-04	K01758	cystathionine gamma-lyase
PP_0894	257.93	4.89	8.78E-04		hypothetical protein PP_0894
PP_0999	1190.65	2.93	8.93E-04	K00926	carbamate kinase
PP_3742	1333.64	2.55	9.12E-04		glutathione S-transferase
PP_0337	1036.23	2.02	9.32E-04		PAS/PAC and GAF sensor-containing diguanylate cyclase/phosphodiesterase
PP_4201	2817.08	3.51	9.36E-04	K03522	electron transfer flavoprotein subunit alpha
PP_0504	3080.95	9.57	9.43E-04	K07275	OmpW family protein
PP_1013	338.77	2.06	9.52E-04		integral membrane sensor signal transduction histidine kinase
PP_3593	241.43	7.77	9.73E-04	K02030	amino acid ABC transporter substrate-binding protein
PP_5139	661.34	3.02	9.80E-04	K01534	heavy metal translocating P-type ATPase
PP_2180	68.82	3.23	9.99E-04		aminotransferase
PP_0738	1727.96	4.70	1.01E-03		hypothetical protein PP_0738
PP_4307	353.14	3.15	1.01E-03		hypothetical protein PP_4307
PP_0619	437.88	6.49	1.02E-03	K01999	branched-chain amino acid ABC transporter substrate-binding protein
PP_3874	1.73	10.33	1.02E-03		hypothetical protein PP_3874
PP_5303	1421.38	5.55	1.03E-03		endoribonuclease
PP_2947	6620.34	2.05	1.12E-03		transcriptional regulator MvaT. P16 subunit
PP_5391	6489.13	4.86	1.15E-03		hypothetical protein PP_5391
PP_1658	939.85	3.33	1.17E-03	K09912	hypothetical protein PP_1658
PP_5183	758.78	8.49	1.23E-03	K01915	glutamine synthetase
PP_4465	318.91	3.16	1.23E-03		porin
PP_4753	706.14	2.58	1.24E-03	K22551	N-methylproline demethylase
PP_3290	239.59	2.87	1.25E-03		universal stress protein
PP_1741	1107.81	3.74	1.27E-03	K02002	glycine betaine ABC transporter substrate-binding protein
PP_2277	1572.87	2.40	1.28E-03		hypothetical protein PP_2277
PP_4478	432.67	4.67	1.31E-03	K06447	succinylglutamic semialdehyde dehydrogenase
PP_1297	6033.89	7.30	1.32E-03	K09969	amino acid ABC transporter substrate-binding protein
PP_4106	134.22	2.05	1.38E-03	K11735	amino acid ABC transporter permease
PP_4086	25.92	8.45	1.38E-03		hypothetical protein PP_4086
PP_4094	60.71	3.59	1.39E-03		hypothetical protein PP_4094
PP_0584	1854.29	3.16	1.41E-03	K03406	methyl-accepting chemotaxis transducer
PP_5278	1379.30	3.98	1.43E-03	K12254	aldehyde dehydrogenase
PP_4865	1564.95	7.28	1.48E-03	K01998	inner-membrane translocator
PP_4350	155.85	2.31	1.49E-03		aminotransferase
PP_3718	48.92	2.53	1.51E-03		hypothetical protein PP_3718
PP_4411	139.85	2.14	1.51E-03		hypothetical protein PP_4411
PP_4459	262.05	2.10	1.55E-03		transposase
PP_1300	1379.04	12.28	1.55E-03	K09972	general amino acid ABC transporter ATP-binding protein
PP_4325	1049.02	2.09	1.61E-03	K02195	heme exporter protein CcmC
PP_2448	494.18	3.11	1.61E-03	K09471	FAD dependent oxidoreductase
PP_4332	948.25	4.52	1.62E-03	K03408	purine-binding chemotaxis protein CheW
PP_4870	1604.97	12.29	1.68E-03		azurin
PP_4659	4596.74	4.76	1.69E-03	K00681	gamma-glutamyltransferase
PP_5313	5342.74	2.97	1.73E-03	K05787	histone family protein DNA-binding protein
PP_4378	53417.77	2.96	1.77E-03	K02406	flagellin FliC
PP_5261	112.66	2.99	1.78E-03		LysR family transcriptional regulator
PP_0246	950.42	2.94	1.78E-03	K07659	osmolarity response regulator
PP_0615	64.69	5.84	1.82E-03	K01996	branched chain amino acid ABC transporter ATP-binding protein
PP_4729	426.72	2.08	1.87E-03	K03631	DNA repair protein RecN
PP_4460	742.05	3.73	1.88E-03		LysR family transcriptional regulator
PP_3881	43.69	4.48	1.89E-03		phage terminase. large subunit
PP_3879	29.78	4.54	1.92E-03		HK97 family phage portal protein
PP_3596	61.90	5.48	1.93E-03		FAD dependent oxidoreductase
PP_5268	130.29	2.55	1.94E-03		Cro/CI family transcriptional regulator
PP_0307	192.08	5.86	1.95E-03		hypothetical protein PP_0307
PP_0585	191.34	2.41	1.98E-03	K19591	MerR family transcriptional regulator
PP_4770	197.09	2.70	1.98E-03	K09945	periplasmic ligand-binding sensor protein
PP_0594	237.57	2.23	2.01E-03	K09017	TetR family transcriptional regulator
PP_4428	512.34	2.35	2.04E-03	K02030	amino acid ABC transporter substrate-binding protein
PP_3949	189.35	2.13	2.04E-03	K22294	TetR family transcriptional regulator
PP_5245	202.69	2.04	2.07E-03		AraC family transcriptional regulator
PP_0368	2935.93	2.74	2.08E-03		acyl-CoA dehydrogenase
PP_0992	19.15	3.29	2.09E-03	K09930	hypothetical protein PP_0992
PP_1307	58.72	4.73	2.10E-03	K03719	AsnC family transcriptional regulator
PP_3492	252.56	3.14	2.12E-03		acyl-CoA dehydrogenase
PP_0975	653.37	3.90	2.18E-03	K03530	histone family protein DNA-binding protein
PP_4991	789.02	2.52	2.22E-03	K02658	response regulator receiver protein
PP_3536	293.69	6.16	2.23E-03		hypothetical protein PP_3536
PP_4615	44.72	2.74	2.27E-03		phosphate-starvation-inducible E
PP_3196	212.76	2.26	2.27E-03		nodulation protein d1
PP_0807	221.42	5.50	2.29E-03	K12266	anaerobic nitric oxide reductase transcriptional regulator
PP_0680	2547.46	3.34	2.30E-03		ATP-dependent protease
PP_2869	33.66	3.28	2.31E-03		NADH:flavin oxidoreductase
PP_0370	775.17	2.49	2.32E-03		acyl-CoA dehydrogenase
PP_5280	43.52	3.85	2.34E-03		hypothetical protein PP_5280
PP_3067	1481.21	2.09	2.34E-03		hypothetical protein PP_3067
PP_4064	414.26	2.10	2.34E-03	K00253	acyl-CoA dehydrogenase
PP_3147	52.97	29.26	2.35E-03	K02055	polyamine ABC transporter substrate-binding protein
PP_0308	247.13	9.39	2.38E-03	K01273	membrane dipeptidase
PP_5378	446.81	3.24	2.42E-03		cytochrome c family protein
PP_5033	64.03	4.18	2.42E-03	K01712	urocanate hydratase
PP_5275	120.29	2.46	2.44E-03		GntR family transcriptional regulator
PP_2643	515.67	3.76	2.44E-03	K03406	methyl-accepting chemotaxis sensory transducer
PP_2310	4533.74	2.86	2.47E-03		methyl-accepting chemotaxis sensory transducer
PP_0658	323.92	3.20	2.51E-03		homocysteine S-methyltransferase
PP_5356	240.07	2.21	2.52E-03		thioesterase
PP_0309	30.13	11.03	2.57E-03		hypothetical protein PP_0309
PP_0328	303.62	2.80	2.57E-03	K00148	formaldehyde dehydrogenase
PP_1206	29391.79	3.11	2.63E-03	K18093	porin
PP_5390	593.32	7.09	2.63E-03		hypothetical protein PP_5390
PP_3910	11.56	2.18	2.64E-03		hypothetical protein PP_3910
PP_3154	2099.93	3.92	2.66E-03	K12976	hypothetical protein PP_3154
PP_4779	1156.04	2.52	2.67E-03	K01241	AMP nucleosidase
PP_0243	558.37	2.28	2.68E-03	K01919	glutamate--cysteine ligase
PP_3291	76.20	2.43	2.70E-03	K07576	metallo-beta-lactamase
PP_4754	312.24	3.16	2.71E-03		TetR family transcriptional regulator
PP_5186	355.62	2.42	2.73E-03	K01438	acetylornithine deacetylase
PP_3236	55.97	2.21	2.77E-03		lipoprotein OprI
PP_5358	1406.29	2.18	2.77E-03		hypothetical protein PP_5358
PP_3839	3857.31	4.16	2.77E-03	K13953	alcohol dehydrogenase
PP_0021	291.21	2.82	2.78E-03		hypothetical protein PP_0021
PP_0227	1880.80	2.98	2.79E-03	K02424	cystine transporter subunit
PP_1139	1492.22	3.35	2.82E-03	K01998	leucine/isoleucine/valine transporter permease subunit
PP_3931	758.07	2.29	2.88E-03		sodium/sulfate symporter family protein
PP_0315	198.21	8.98	2.91E-03	K00479	Rieske (2Fe-2S) domain-containing protein
PP_4621	81.53	6.58	2.91E-03	K00451	homogentisate 1.2-dioxygenase
PP_0112	1845.32	3.25	2.92E-03	K02073	metal ABC transporter substrate-binding protein
PP_1465	2780.24	2.96	2.95E-03	K02884	50S ribosomal protein L19
PP_4352	213.68	3.26	2.97E-03	K02401	flagellar biosynthesis protein FlhB
PP_1116	126.17	2.14	2.98E-03		resolvase site-specific recombinase
PP_0617	88.83	6.58	3.02E-03	K01998	inner-membrane translocator
PP_3901	129.47	2.75	3.04E-03		hypothetical protein PP_3901
PP_0217	387.45	2.15	3.06E-03		fatty acid desaturase
PP_4863	1083.74	9.83	3.06E-03	K01996	branched chain amino acid ABC transporter ATP-binding protein
PP_4454	184.23	2.54	3.07E-03	K02034	opine ABC transporter permease
PP_0339	4287.45	5.99	3.09E-03	K00163	pyruvate dehydrogenase subunit E1
PP_0094	146.54	2.06	3.11E-03	K01091	HAD superfamily hydrolase
PP_4578	71.77	2.25	3.12E-03		major facilitator family transporter
PP_5118	1639.32	2.32	3.14E-03	K01011	rhodanese domain-containing protein
PP_3732	193.60	2.65	3.14E-03		enoyl-CoA hydratase
PP_4251	94.46	46.03	3.15E-03	K00405	cbb3-type cytochrome c oxidase subunit II
PP_0313	51.68	2.76	3.16E-03	K03521	electron transfer flavoprotein subunit beta
PP_0325	363.46	3.38	3.17E-03	K00302	sarcosine oxidase subunit alpha
PP_5208	497.89	4.41	3.20E-03	K01992	ABC transporter
PP_0065	712.84	2.23	3.21E-03	K03499	potassium transporter peripheral membrane protein
PP_5206	1226.01	3.19	3.22E-03	K01993	secretion protein HlyD family protein
PP_3710	158.49	3.17	3.22E-03		hypothetical protein PP_3710
PP_1787	5108.59	3.45	3.23E-03		hypothetical protein PP_1787
PP_0272	70.38	6.02	3.24E-03	K16088	TonB-dependent siderophore receptor
PP_0614	163.48	3.06	3.30E-03	K06016	allantoate amidohydrolase
PP_0173	811.28	3.30	3.31E-03		transcriptional factor-like protein
PP_0055	147.11	2.28	3.32E-03		LysR family transcriptional regulator
PP_5029	185.01	2.28	3.32E-03	K01458	N-formylglutamate amidohydrolase
PP_0311	167.59	4.31	3.32E-03	K21834	(Fe-S)-binding protein
PP_5340	49.01	7.68	3.33E-03		histone deacetylase superfamily protein
PP_4480	910.17	3.68	3.33E-03	K00673	arginine N-succinyltransferase subunit alpha
PP_0620	276.71	6.06	3.34E-03		GntR family transcriptional regulator
PP_3662	3284.18	3.74	3.37E-03	K06966	decarboxylase
PP_3080	228.88	16.21	3.41E-03	K01626	phospho-2-dehydro-3-deoxyheptonate aldolase
PP_3699	832.67	2.58	3.41E-03		hypothetical protein PP_3699
PP_3591	288.60	2.30	3.42E-03	K13609	malate/L-lactate dehydrogenase
PP_3680	1292.14	2.00	3.42E-03	K07459	hypothetical protein PP_3680
PP_4427	110.37	2.73	3.42E-03	K02029	amino acid ABC transporter permease
PP_3948	78.59	15.14	3.44E-03	K18030	aldehyde dehydrogenase
PP_1453	754.58	2.05	3.45E-03		NUDIX hydrolase
PP_0661	62.17	2.21	3.54E-03	K03566	transcriptional regulator AmpR
PP_1705	16.44	2.09	3.56E-03	K00362	nitrite reductase (NAD(P)H) large subunit
PP_0428	863.16	4.39	3.60E-03	K02503	histidine triad (HIT) protein
PP_3878	23.22	4.11	3.61E-03		phage minor capsid protein C
PP_0829	68.74	2.04	3.64E-03		hypothetical protein PP_0829
PP_4929	46.42	3.09	3.66E-03		LysR family transcriptional regulator
PP_0482	7561.24	2.02	3.66E-03	K03594	bacterioferritin
PP_3736	141.40	4.37	3.67E-03	K03862	Rieske (2Fe-2S) domain-containing protein
PP_3179	205.67	3.67	3.70E-03		LysR family transcriptional regulator
PP_0299	627.75	5.43	3.71E-03		hypothetical protein PP_0299
PP_1389	420.00	2.21	3.71E-03	K01003	carboxyphosphonoenolpyruvate phosphonomutase
PP_4573	399.79	2.75	3.73E-03		ATPase AAA
PP_4464	42.55	2.38	3.73E-03		LysR family transcriptional regulator
PP_1009	1079.11	3.55	3.73E-03	K00134	glyceraldehyde-3-phosphate dehydrogenase. type I
PP_4253	249.55	30.16	3.73E-03	K00406	cytochrome c oxidase. cbb3-type subunit III
PP_0274	346.72	2.18	3.75E-03	K18831	helix-turn-helix domain-containing protein
PP_1010	578.99	4.64	3.76E-03	K01690	phosphogluconate dehydratase
PP_0318	42.97	13.81	3.77E-03		hypothetical protein PP_0318
PP_1173	259.10	5.30	3.77E-03		porin
PP_3772	362.68	2.16	3.80E-03		phage repressor
PP_3079	247.93	2.63	3.84E-03	K03769	peptidyl-prolyl cis-trans isomerase
PP_4905	716.11	2.43	3.84E-03	K02556	flagellar motor protein MotA
PP_0306	79.05	3.49	3.85E-03		hypothetical protein PP_0306
PP_4458	239.20	2.02	3.85E-03	K02035	opine ABC transporter substrate-binding protein
PP_0597	1887.54	9.86	3.86E-03	K00140	methylmalonate-semialdehyde dehydrogenase
PP_1366	13082.74	2.33	3.88E-03		transcriptional regulator MvaT. P16 subunit
PP_0510	1525.84	2.25	3.89E-03	K05838	thioredoxin
PP_4367	386.06	2.21	3.93E-03	K02411	flagellar assembly protein H
PP_4399	704.72	3.01	3.95E-03	K01915	glutamine synthetase
PP_4290	123.92	4.94	3.96E-03	K24206	xanthine/uracil permease
PP_4633	386.03	3.73	3.97E-03	K09948	hypothetical protein PP_4633
PP_4400	327.10	2.83	3.98E-03	K03719	transcriptional regulator BkdR
PP_0018	941.40	3.73	3.98E-03		hypothetical protein PP_0018
PP_4182	7385.51	3.97	3.99E-03		hypothetical protein PP_4182
PP_5184	1970.37	2.71	3.99E-03	K01915	glutamine synthetase
PP_1012	151.41	4.53	4.01E-03		winged helix family two component transcriptional regulator

Gene ID	baseMean (normalized)	log2 Fold Change	p- value	KO term	Description
PP_3235	24.23	4.58	4.04E-03		hypothetical protein PP_3235
PP_3715	154.91	124.68	4.07E-03	K01856	muconate and chloromuconate cycloisomerase
PP_0618	70.70	10.29	4.09E-03	K01997	inner-membrane translocator
PP_0298	3434.82	2.82	4.12E-03	K21826	AraC family transcriptional regulator
PP_2439	743.09	4.28	4.14E-03	K24119	alkyl hydroperoxide reductase
PP_1362	1392.58	2.76	4.17E-03	K00873	pyruvate kinase
PP_0915	11511.60	2.55	4.20E-03	K04564	superoxide dismutase
PP_4187	8079.51	2.74	4.22E-03	K00382	dihydrolipoamide dehydrogenase
PP_1725	256.04	2.39	4.22E-03		type I phosphodiesterase/nucleotide pyrophosphatase
PP_3511	4248.66	2.24	4.23E-03	K00826	branched-chain amino acid aminotransferase
PP_4842	12.93	3.28	4.29E-03	K11960	branched-chain amino acid ABC transporter permease
PP_4841	113.94	3.12	4.36E-03	K11959	branched-chain amino acid ABC transporter substrate-binding protein
PP_5181	8069.41	3.03	4.37E-03	K11073	spermidine/putrescine ABC transporter substrate-binding protein
PP_4377	757.23	2.91	4.37E-03	K06603	flagellin FlaG
PP_1773	566.84	4.23	4.40E-03	K05788	integration host factor subunit beta
PP_3654	51.47	4.63	4.43E-03		AsnC family transcriptional regulator
PP_0295	361.32	5.69	4.44E-03	K02001	glycine betaine/L-proline ABC transporter permease
PP_0481	371.89	2.16	4.47E-03	K03781	catalase
PP_0198	2054.72	2.57	4.48E-03	K05834	amino acid transporter LysE
PP_4888	2194.02	4.43	4.53E-03		methyl-accepting chemotaxis sensory transducer
PP_4716	2089.22	2.04	4.54E-03	K03431	phosphoglucosamine mutase
PP_0300	353.54	2.48	4.54E-03		metallopeptidase
PP_1169	146.48	5.04	4.55E-03		TRAP dicarboxylate transporter subunit DctP
PP_1298	875.42	11.42	4.57E-03	K09970	polar amino acid ABC transporter permease
PP_1021	121.85	2.22	4.58E-03	K19337	DNA-binding transcriptional regulator HexR
PP_4450	292.37	2.02	4.59E-03		phosphoglycerate mutase
PP_4357	402.09	2.67	4.64E-03	K02417	flagellar motor switch protein
PP_4461	155.37	2.11	4.65E-03		major facilitator family transporter
PP_1138	717.73	4.33	4.69E-03	K01995	leucine/isoleucine/valine transporter ATP-binding subunit
PP_0093	333.94	2.34	4.70E-03		hypothetical protein PP_0093
PP_4620	102.86	3.14	4.72E-03	K01555	fumarylacetoacetase
PP_2010	299.69	2.25	4.73E-03	K12262	cytochrome B561
PP_3148	159.22	10.23	4.80E-03	K01915	glutamine synthetase
PP_4412	194.87	2.74	4.81E-03		hypothetical protein PP_4412
PP_4479	466.71	4.38	4.85E-03	K00673	arginine N-succinyltransferase subunit beta
PP_0181	1952.43	2.85	4.85E-03		hypothetical protein PP_0181
PP_0421	456.90	3.03	4.88E-03	K00766	anthranilate phosphoribosyltransferase
PP_5180	660.71	4.15	4.96E-03	K11073	spermidine/putrescine ABC transporter substrate-binding protein
PP_1788	1461.75	4.92	4.98E-03		hypothetical protein PP_1788
PP_0765	8456.44	2.23	4.99E-03		hypothetical protein PP_0765
PP_0312	29.39	4.97	5.00E-03	K03522	electron transfer flavoprotein subunit alpha
PP_3739	406.76	2.38	5.00E-03	K11475	GntR family transcriptional regulator
PP_4250	468.92	18.95	5.01E-03	K00404	cbb3-type cytochrome c oxidase subunit I
PP_4338	3048.03	2.42	5.04E-03	K03407	chemotaxis protein CheA
PP_3875	3.46	15.36	5.05E-03		hypothetical protein PP_3875
PP_0283	666.31	2.72	5.06E-03	K23058	amino acid ABC transporter ATP-binding protein
PP_4843	10.59	2.40	5.09E-03	K11961	branched-chain amino acid ABC transporter permease
PP_5165	243.46	2.64	5.13E-03	K02073	NLPA lipoprotein
PP_4483	620.44	2.82	5.14E-03	K10025	basic amino acid ABC transporter ATP-binding protein
PP_3332	25.18	2.75	5.16E-03		cytochrome c-type protein
PP_4867	8269.71	8.42	5.17E-03	K01999	ABC transporter substrate-binding protein
PP_1464	6679.28	2.68	5.20E-03	K00554	tRNA (guanine-N(1)-)-methyltransferase
PP_4104	507.73	2.76	5.21E-03	K11209	glutathione S-transferase
PP_0400	113.31	2.01	5.22E-03	K06195	ApaG protein
PP_0122	1254.53	2.47	5.24E-03		hypothetical protein PP_0122
PP_4229	25.87	2.23	5.26E-03		hypothetical protein PP_4229
PP_4354	94.88	2.28	5.27E-03	K02420	flagellar biosynthesis protein FliQ
PP_5388	170.86	2.67	5.27E-03	K07810	hypothetical protein PP_5388
PP_3714	105.68	138.13	5.28E-03	K03464	muconolactone isomerase
PP_3871	3.70	11.98	5.34E-03		hypothetical protein PP_3871
PP_0779	463.70	2.30	5.36E-03	K03406	methyl-accepting chemotaxis transducer/sensory box protein
PP_0285	151.27	3.99	5.40E-03		hypothetical protein PP_0285
PP_1011	544.65	4.62	5.42E-03	K00845	glucokinase
PP_0884	430.90	3.49	5.44E-03	K12368	peptide ABC transporter substrate-binding protein
PP_4348	37.43	2.67	5.48E-03	K01760	cystathionine beta-lyase
PP_3180	72.48	3.01	5.55E-03		SMP-30/gluconolaconase/LRE domain-containing protein
PP_5267	9940.33	2.92	5.59E-03		cytochrome c5
PP_4022	694.46	2.71	5.61E-03		hypothetical protein PP_4022
PP_2600	102.27	2.18	5.63E-03		FAD-binding dehydrogenase
PP_2601	45.37	2.80	5.63E-03		IclR family transcriptional regulator
PP_5324	1574.69	3.01	5.63E-03		response regulator receiver protein
PP_4289	364.61	4.83	5.68E-03		hypothetical protein PP_4289
PP_4486	2312.18	4.28	5.68E-03	K10022	amino acid ABC transporter substrate-binding protein
PP_3960	192.59	2.05	5.69E-03		TetR family transcriptional regulator
PP_0911	13.73	2.99	5.71E-03		hypothetical protein PP_0911
PP_0895	23.31	2.76	5.71E-03		hypothetical protein PP_0895
PP_0077	77.57	2.29	5.77E-03	K01133	choline sulfatase
PP_4982	1008.42	3.18	5.82E-03		cytochrome B561
PP_1091	673.97	3.20	5.86E-03		hypothetical protein PP_1091
PP_3877	10.42	18.39	5.86E-03		hypothetical protein PP_3877
PP_5362	145.49	2.24	5.87E-03		hypothetical protein PP_5362
PP_4983	942.62	2.65	5.91E-03	K00274	amine oxidase
PP_0917	89.71	2.02	5.94E-03	K05596	chromosome replication initiation inhibitor protein
PP_1869	126.01	4.18	5.96E-03	K15777	hypothetical protein PP_1869
PP_1699	19.85	2.63	6.00E-03		amidohydrolase
PP_1002	4197.53	4.39	6.00E-03	K03758	arginine/ornithine antiporter
PP_4864	1425.80	5.99	6.02E-03	K01995	branched chain amino acid ABC transporter ATP-binding protein
PP_4451	460.81	3.95	6.04E-03		hypothetical protein PP_4451
PP_0596	5765.11	4.81	6.04E-03	K00822	beta alanine--pyruvate transaminase
PP_3557	327.12	2.29	6.06E-03	K03406	methyl-accepting chemotaxis transducer

Gene ID	baseMean (normalized)	log2 Fold Change	p- value	KO term	Description
PP_5364	361.93	2.68	6.07E-03	K06131	cardiolipin synthetase
PP_5020	2274.81	5.11	6.09E-03	K03406	methyl-accepting chemotaxis sensory transducer
PP_1137	2902.26	2.71	6.14E-03	K01996	branched chain amino acid ABC transporter ATP-binding protein
PP_5138	310.51	2.02	6.15E-03		LysR family transcriptional regulator
PP_0463	921.68	4.22	6.16E-03	K02961	30S ribosomal protein S17
PP_4249	518.03	6.36	6.19E-03	K07020	hypothetical protein PP_4249
PP_4186	15123.14	3.26	6.21E-03	K01903	succinyl-CoA synthetase subunit beta
PP_1514	157.11	2.05	6.29E-03		hypothetical protein PP_1514
PP_0595	96.63	2.21	6.29E-03	K21699	LysR family transcriptional regulator
PP_4456	153.46	2.92	6.29E-03		nopaline dehydrogenase
PP_3947	18.85	9.29	6.30E-03	K18029	(2Fe-2S)-binding protein
PP_0459	3153.94	2.38	6.34E-03	K02890	50S ribosomal protein L22
PP_0336	289.55	3.92	6.34E-03	K07304	methionine sulfoxide reductase A
PP_3491	210.14	4.57	6.35E-03		enoly-coenzyme A hydratase/isomerase
PP_4273	134.58	2.42	6.35E-03		MerR family transcriptional regulator
PP_1001	2879.38	6.64	6.38E-03	K01478	arginine deiminase
PP_5381	43.23	2.06	6.40E-03		hypothetical protein PP_5381
PP_4999	389.02	2.17	6.40E-03	K01465	dihydroorotase
PP_3713	944.24	95.94	6.40E-03	K03381	catechol 1.2-dioxygenase
PP_3277	2.66	4.44	6.40E-03	K02610	phenylacetate-CoA oxygenase subunit PaaB
PP_0079	140.03	4.31	6.42E-03		LysR family transcriptional regulator
PP_16SD	0.60	8.19	6.45E-03		amidohydrolase
PP_5217	1368.20	2.02	6.46E-03	K00937	polyphosphate kinase
PP_3406	65.55	2.14	6.51E-03		acetyltransferase
PP_1022	812.64	4.21	6.51E-03	K00036	glucose-6-phosphate 1-dehydrogenase
PP_0369	236.51	3.04	6.52E-03		diguanylate cyclase
PP_0326	75.37	2.55	6.56E-03	K00305	sarcosine oxidase subunit gamma
PP_4425	148.55	2.93	6.57E-03	K02028	amino acid ABC transporter ATP-binding protein
PP_0296	1756.85	4.09	6.57E-03	K02002	glycine/betaine ABC transporter substrate-binding protein
PP_4147	454.70	2.36	6.60E-03	K13893	peptide ABC transporter substrate-binding protein
PP_2077	33.28	3.77	6.66E-03	K01586	diaminopimelate decarboxylase
PP_0251	711.49	3.13	6.67E-03		hypothetical protein PP_0251
PP_0858	676.06	2.15	6.70E-03	K14287	aminotransferase
PP_0377	67.65	2.08	6.72E-03	K06138	pyrroloquinoline quinone biosynthesis protein PqqD
PP_5016	369.84	3.81	6.73E-03	K03116	twin-arginine translocation protein TatA
PP_0488	72.95	3.15	6.77E-03	K16066	short chain dehydrogenase/reductase oxidoreductase
PP_4423	69.96	2.13	6.79E-03	K15784	hypothetical protein PP_4423
PP_2638	64.01	2.41	6.80E-03	K20543	cellulose synthase subunit BcsC
PP_4481	1642.98	3.09	6.84E-03	K00840	bifunctional N-succinyldiaminopimelate-aminotransferase/acetylornithine transaminase
PP_5182	947.19	10.27	6.86E-03	K12256	aminotransferase
PP_2950	382.40	2.59	6.89E-03	K09958	hypothetical protein PP_2950
PP_2695	31.40	2.12	6.93E-03		LysR family transcriptional regulator
PP_0739	1172.43	2.64	6.94E-03	K01669	deoxyribodipyrimidine photo-lyase
PP_1141	18476.24	2.88	7.00E-03	K01999	ABC transporter substrate-binding protein
PP_1170	44.45	3.59	7.07E-03		SMP-30/gluconolaconase/LRE domain-containing protein
PP_4305	524.91	2.41	7.11E-03	K02048	sulfate ABC transporter substrate-binding protein
PP_4452	72.18	2.97	7.14E-03	K04940	NAD/NADP octopine/nopaline dehydrogenase
PP_0470	1403.50	2.25	7.24E-03	K02881	50S ribosomal protein L18
PP_3537	215.41	27.66	7.33E-03	K00481	4-hydroxybenzoate 3-monooxygenase
PP_4433	42.11	4.13	7.38E-03		amino acid MFS transporter
PP_0323	77.50	3.78	7.39E-03	K00303	sarcosine oxidase subunit beta
PP_1228	41.41	5.05	7.41E-03	K03406	methyl-accepting chemotaxis transducer
PP_1645	81.20	3.48	7.45E-03	K00537	arsenate reductase
PP_3883	5.25	4.44	7.45E-03		phage holin
PP_4179	1179.96	2.59	7.48E-03	K04079	heat shock protein 90
PP_3885	622.63	2.81	7.51E-03		hypothetical protein PP_3885
PP_4365	151.27	2.14	7.53E-03	K02413	flagellar biosynthesis chaperone
PP_4668	148.57	2.10	7.54E-03		LysR family transcriptional regulator
PP_1400	99.06	98.28	7.57E-03	K03761	metabolite/H+ symportermajor facilitator superfamily metabolite/H+ symporter
PP_3898	134.42	2.74	7.62E-03		hypothetical protein PP_3898
PP_3594	86.78	2.69	7.65E-03	K02029	polar amino acid ABC transporter inner membrane subunit
PP_5339	207.96	8.56	7.67E-03		AraC family transcriptional regulator
PP_3863	8.54	3.40	7.68E-03		phage tail protein
PP_4315	105.61	3.18	7.68E-03		PhzF family phenazine biosynthesis protein
PP_1721	890.19	2.17	7.71E-03		HAD superfamily hydrolase
PP_0321	731.45	3.94	7.75E-03	K01620	threonine aldolase
PP_4351	24.79	2.33	7.80E-03		aminotransferase
PP_4337	1607.61	2.61	7.85E-03	K03412	chemotaxis-specific methylesterase
PP_5271	230.57	2.24	7.92E-03	K03719	leucine-responsive regulatory protein
PP_3353	87.72	6.05	8.01E-03	K13444	hypothetical protein PP_3353
PP_4293	66.51	3.29	8.07E-03		hypothetical protein PP_4293
PP_1360	716.45	2.91	8.07E-03	K04078	co-chaperonin GroES
PP_3819	1475.21	2.38	8.11E-03	K00383	glutathione reductase
PP_3122	97.11	2.10	8.13E-03	K01028	3-oxoacid CoA-transferase subunit A
PP_2565	140.75	2.06	8.14E-03		UvrD/REP helicase
PP_0038	26.22	3.57	8.14E-03		hypothetical protein PP_0038
PP_5233	173.69	2.04	8.30E-03	K03320	ammonium transporter
PP_3897	420.39	2.12	8.30E-03		hypothetical protein PP_3897
PP_0636	144.88	3.49	8.32E-03		cold shock DNA-binding domain-containing protein
PP_0268	706.79	3.10	8.40E-03		porin
PP_1592	1631.43	2.71	8.41E-03	K02357	elongation factor Ts
PP_3186	90.84	2.03	8.44E-03	K03707	TenA family transcriptional activator
PP_3352	220.70	10.54	8.45E-03		arylsulfatase
PP_2603	123.33	4.35	8.46E-03		xylose isomerase
PP_2194	104.12	2.54	8.47E-03		LysR family transcriptional regulator
PP_4752	201.57	2.02	8.47E-03		aminopeptidase
PP_0226	405.09	2.42	8.51E-03	K10009	polar amino acid ABC transporter inner membrane subunit
PP_5379	751.36	3.68	8.54E-03	K07233	copper resistance B
PP_0013	2028.30	2.12	8.55E-03	K02470	DNA gyrase subunit B
PP_5409	841.14	2.75	8.57E-03	K00820	glucosamine--fructose-6-phosphate aminotransferase
PP_4268	4432.42	2.28	8.68E-03	K09747	hypothetical protein PP_4268

Gene ID	baseMean (normalized)	log2 Fold Change	p- value	KO term	Description
PP_4202	2164.12	3.21	8.73E-03	K03521	electron transfer flavoprotein subunit beta
PP_1142	1087.77	2.77	8.76E-03		hypothetical protein PP_1142
PP_5046	2996.01	3.67	8.80E-03	K01915	glutamine synthetase. type I
PP_3289	30.42	5.79	8.84E-03		acetyltransferase
PP_0563	1301.09	2.26	8.88E-03		response regulator receiver modulated diguanylate cyclase
PP_4242	88.10	3.40	8.90E-03		hypothetical protein PP_4242
PP_0451	13310.89	2.59	9.02E-03	K02355	elongation factor G
PP_4144	551.10	2.08	9.04E-03	K01069	hydroxyacylglutathione hydrolase
PP_4981	9018.57	2.99	9.16E-03		hypothetical protein PP_4981
PP_1452	397.60	2.39	9.22E-03		NUDIX hydrolase
PP_0145	392.71	4.82	9.27E-03	K03324	sodium-dependent inorganic phosphate (Pi) transporter
PP_3174	524.55	2.26	9.28E-03		aldo/keto reductase
PP_0228	829.07	2.17	9.28E-03	K00640	serine O-acetyltransferase. partial
PP_3730	98.71	2.60	9.30E-03		transcriptional regulator
PP_1211	148.84	2.07	9.37E-03		hypothetical protein PP_1211
PP_3700	762.50	2.17	9.42E-03		hypothetical protein PP_3700
PP_4751	118.96	2.01	9.47E-03	K02028	amino acid ABC transporter ATP-binding protein
PP_0297	37.88	4.24	9.48E-03	K01752	L-serine dehydratase
PP_4259	1118.66	2.59	9.52E-03		(Fe-S)-binding protein
PP_0721	3501.00	3.48	9.57E-03	K02897	50S ribosomal protein L25
PP_1157	42.40	2.22	9.61E-03	K01652	acetolactate synthase
PP_4232	139.21	7.33	9.61E-03		gluconate 2-dehydrogenase
PP_4429	603.21	3.26	9.63E-03		GntR family transcriptional regulator
PP_3597	120.34	5.31	9.63E-03	K02028	amino acid ABC transporter ATP-binding protein
PP_2155	107.96	2.21	9.71E-03	K09810	lipoprotein releasing system. ATP-binding protein
PP_3902	17.52	2.46	9.77E-03		hypothetical protein PP_3902
PP_2648	253.66	22.39	9.81E-03		universal stress protein
PP_5346	1933.46	2.19	9.82E-03	K01960	pyruvate carboxylase subunit B
PP_0324	27.60	3.48	9.87E-03	K00304	sarcosine oxidase subunit delta
PP_4380	1595.92	2.08	9.95E-03	K02397	flagellar hook-associated protein FlgL
PP_4424	398.19	2.61	9.99E-03	K03719	AsnC family transcriptional regulator
PP_1922	342.46	3.88	1.00E-02		hypothetical protein PP_1922
PP_1662	353.93	2.16	1.00E-02		hypothetical protein PP_1662
PP_3161	48913.27	2.02	1.01E-02	K05549	benzoate dioxygenase subunit alpha
PP_5196	203.37	2.04	1.01E-02	K02012	iron ABC transporter substrate-binding protein
PP_2206	51.78	2.16	1.02E-02	K08303	U32 family peptidase
PP_4869	789.49	2.44	1.02E-02	K01916	NAD synthetase
PP_4538	210.39	4.78	1.04E-02	K01118	NAD(P)H dehydrogenase (quinone)
PP_0750	1231.37	3.28	1.04E-02		hypothetical protein PP_0750
PP_0367	2051.80	2.38	1.04E-02		hypothetical protein PP_0367
PP_4904	785.51	2.63	1.04E-02	K02557	flagellar motor protein MotB
PP_5408	84.60	2.34	1.04E-02		hypothetical protein PP_5408
PP_0446	5796.85	3.80	1.05E-02	K02935	50S ribosomal protein L7/L12
PP_1722	227.50	2.06	1.05E-02	K02052	ABC transporter ATP-binding protein
PP_5095	295.91	2.00	1.06E-02	K00286	pyrroline-5-carboxylate reductase
PP_5114	229.00	2.10	1.07E-02	K08316	hypothetical protein PP_5114
PP_0314	217.73	2.03	1.08E-02		hypothetical protein PP_0314
PP_5215	6928.73	2.05	1.08E-02	K03671	thioredoxin
PP_4457	155.34	2.37	1.09E-02		nopaline dehydrogenase
PP_1363	279.03	2.70	1.10E-02		hypothetical protein PP_1363
PP_4432	373.13	2.21	1.10E-02	K15783	peptidase. M24 family protein
PP_5128	2162.80	2.02	1.10E-02	K01687	dihydroxy-acid dehydratase
PP_4151	261.89	2.87	1.11E-02		excisionase
PP_1309	1043.46	2.80	1.11E-02	K09908	hypothetical protein PP_1309
PP_ICE67800	51.55	2.28	1.11E-02	K02116	PP_aacC1
PP_0319	62.17	23.77	1.12E-02	K09888	hypothetical protein PP_0319
PP_0134	90.91	2.04	1.13E-02	K04065	transport-associated protein
PP_4455	356.07	3.30	1.13E-02	K02033	opine ABC transporter substrate-binding protein
PP_4375	2503.45	2.89	1.13E-02	K02422	flagellar protein FliS
PP_0782	26.13	2.36	1.14E-02		hypothetical protein PP_0782
PP_1024	371.64	13.84	1.17E-02	K01625	keto-hydroxyglutarate-aldolase/keto-deoxy-phosphogluconate aldolase
PP_3749	13.22	2.02	1.18E-02		hypothetical protein PP_3749
PP_4288	241.20	2.59	1.19E-02	K01483	ureidoglycolate hydrolase
PP_0637	41.13	2.12	1.19E-02		ISPpu15. transposase Orf2
PP_3823	147.57	15.16	1.19E-02		cytochrome c-type protein
PP_0322	134.00	7.08	1.21E-02	K00600	serine hydroxymethyltransferase
PP_4339	1079.09	2.59	1.22E-02	K03414	chemotaxis protein CheZ
PP_4349	155.97	2.04	1.23E-02		hypothetical protein PP_4349
PP_0422	431.22	2.84	1.23E-02	K01609	indole-3-glycerol phosphate synthase
PP_0371	189.09	3.63	1.24E-02		LysR family transcriptional regulator
PP_0050	198.68	2.14	1.26E-02		hypothetical protein PP_0050
PP_3952	1526.49	2.25	1.27E-02	K01032	3-oxoadipate CoA-transferase subunit B
PP_1530	612.99	2.26	1.28E-02	K00674	2.3.4.5-tetrahydropyridine-2.6-carboxylate N-succinyltransferase
PP_3508	176.85	2.65	1.30E-02	K02234	cobalamin biosynthesis protein CobW
PP_4291	61.14	2.57	1.31E-02		hypothetical protein PP_4291
PP_4227	35.69	2.35	1.31E-02		hypothetical protein PP_4227
PP_3869	35.79	3.92	1.33E-02		phage sheath protein
PP_4494	70.11	2.46	1.34E-02		LysR family transcriptional regulator
PP_2564	428.53	2.11	1.34E-02	K06911	pirin
PP_0152	1194.59	3.49	1.34E-02		hypothetical protein PP_0152
PP_4764	71.38	2.12	1.36E-02		histone deacetylase superfamily protein
PP_4447	2301.85	2.13	1.36E-02		hypothetical protein PP_4447
PP_4287	242.25	3.91	1.36E-02		hypothetical protein PP_4287
PP_3712	165.58	7.50	1.37E-02		hypothetical protein PP_3712
PP_3961	66.37	3.64	1.37E-02		major facilitator superfamily transporter
PP_4323	100.86	4.66	1.38E-02	K02197	cytochrome C biogenesis protein CcmE
PP_4619	114.48	3.55	1.38E-02	K01800	maleylacetoacetate isomerase
PP_1023	147.04	14.77	1.38E-02	K01057	6-phosphogluconolactonase
PP_3737	148.10	2.69	1.39E-02	K03863	ferredoxin
PP_3773	156.81	5.94	1.39E-02		hypothetical protein PP_3773
PP_2903	1117.22	2.22	1.39E-02	K03768	peptidyl-prolyl cis-trans isomerase B
PP_4435	99.64	2.17	1.39E-02		hypothetical protein PP_4435

Gene ID	baseMean (normalized)	log2 Fold Change	p- value	KO term	Description
PP_5179	1434.74	2.57	1.40E-02	K11076	spermidine/putrescine ABC transporter ATPase
PP_0467	240.00	5.95	1.40E-02	K02954	30S ribosomal protein S14
PP_5162	96.16	2.19	1.40E-02		hypothetical protein PP_5162
PP_0625	3454.27	3.39	1.41E-02	K03695	ATP-dependent Clp protease. ATP-binding subunit ClpB
PP_5289	964.73	2.06	1.41E-02	K00930	acetylglutamate kinase
PP_2543	75.78	2.84	1.42E-02	K11735	amino acid ABC transporter permease
PP_5211	915.92	2.27	1.42E-02	K07232	ChaC family protein
PP_1223	6092.81	2.92	1.43E-02	K03640	peptidoglycan-associated lipoprotein OprL
PP_2407	59.58	2.57	1.43E-02	K03786	3-dehydroquinate dehydratase
PP_3349	126.46	2.07	1.44E-02	K05819	3-hydroxyphenylpropionic transporter MhpT
PP_4974	116.69	3.96	1.44E-02	K03316	sodium/hydrogen exchanger
PP_4463	47.60	2.68	1.46E-02	K11206	carbon-nitrogen hydrolase
PP_0797	99.98	2.48	1.46E-02		hypothetical protein PP_0797
PP_0789	289.34	2.04	1.46E-02	K03806	N-acetyl-anhydromuranmyl-L-alanine amidase
PP_0883	143.95	3.43	1.46E-02		porin
PP_3822	254.83	18.97	1.48E-02	K19713	cytochrome c family protein
PP_2298	685.87	2.05	1.49E-02		hypothetical protein PP_2298
PP_3828	75.12	3.37	1.50E-02	K02020	molybdenum ABC transporter substrate-binding protein
PP_0490	369.19	2.41	1.50E-02	K00124	formate dehydrogenase subunit beta
PP_2114	13.26	2.08	1.51E-02		ISPpu8. transposase
PP_5392	4716.58	2.11	1.51E-02		hypothetical protein PP_5392
PP_0034	20.23	2.15	1.52E-02	K20534	ribonuclease III
PP_0586	719.89	3.89	1.52E-02	K17686	heavy metal translocating P-type ATPase
PP_5344	42.26	4.05	1.52E-02		acetyltransferase
PP_5314	354.43	2.02	1.54E-02	K05297	FAD-dependent pyridine nucleotide-disulfide oxidoreductase
PP_4664	21.01	2.17	1.55E-02		LysR family transcriptional regulator
PP_0744	1162.01	2.19	1.56E-02	K01772	ferrochelatase
PP_3766	184.94	2.36	1.56E-02	K01759	lactoylglutathione lyase
PP_0335	372.15	2.47	1.58E-02		glutathione S-transferase
PP_0452	9892.57	3.78	1.58E-02	K02358	elongation factor Tu
PP_1361	3564.73	4.01	1.58E-02	K04077	molecular chaperone GroEL
PP_1637	1894.66	2.95	1.60E-02		LysR family transcriptional regulator
PP_3983	612.54	2.09	1.60E-02		hypothetical protein PP_3983
PP_3873	6.03	13.08	1.61E-02		hypothetical protein PP_3873
PP_3862	5.52	2.76	1.62E-02		phage assembly protein
PP_3860	20.66	2.48	1.63E-02		phage FluMu protein gp47
PP_1774	757.80	2.04	1.63E-02		hypothetical protein PP_1774
PP_2807	15.56	2.78	1.66E-02		hypothetical protein PP_2807
PP_1985	894.33	2.30	1.66E-02	K01703	isopropylmalate isomerase large subunit
PP_4756	288.40	3.07	1.68E-02	K03293	amino acid permease
PP_5015	353.12	2.19	1.68E-02	K01523	phosphoribosyl-ATP pyrophosphatase
PP_5246	217.08	2.25	1.68E-02	K11747	potassium efflux system protein
PP_0897	1918.41	2.45	1.70E-02	K01676	Fe-S type. tartrate/fumarate subfamily hydro-lyase subunit alpha
PP_1140	951.89	2.95	1.70E-02	K01997	inner-membrane translocator
PP_3891	4.62	2.49	1.71E-02		hypothetical protein PP_3891
PP_1136	172.78	3.18	1.71E-02		hypothetical protein PP_1136
PP_2553	2669.40	2.07	1.74E-02		major facilitator family transporter
PP_2844	2.25	3.48	1.76E-02	K01429	urease subunit beta
PP_0190	28.48	2.10	1.76E-02	K03611	disulfide bond formation protein DsbB
PP_4790	465.15	2.02	1.78E-02	K03820	apolipoprotein N-acyltransferase
PP_0267	168.00	3.02	1.79E-02	K02014	TonB-dependent siderophore receptor
PP_1697	56.77	2.51	1.80E-02	K03710	GntR family transcriptional regulator
PP_0465	3229.90	2.90	1.83E-02	K02895	50S ribosomal protein L24
PP_4462	25.23	3.29	1.84E-02		4-hydroxy-4-methyl-2-oxoglutarate aldolase
PP_1229	81.31	6.36	1.84E-02	K14052	amino acid ABC transporter permease
PP_3237	37.29	2.40	1.85E-02		universal stress protein
PP_1810	34.82	2.01	1.86E-02		hypothetical protein PP_1810
PP_4975	1539.01	2.31	1.86E-02		thioesterase
PP_0234	2757.94	2.46	1.86E-02		porin
PP_0951	29162.86	3.94	1.87E-02	K05808	sigma 54 modulation protein/ribosomal protein S30EA
PP_4194	6462.15	2.32	1.89E-02	K01647	type II citrate synthase
PP_3460	9.81	3.74	1.90E-02		hypothetical protein PP_3460
PP_0273	10.52	16.53	1.95E-02		hypothetical protein PP_0273
PP_3234	43.68	13.38	1.96E-02	K13993	heat shock protein 20
PP_5030	113.66	3.20	1.98E-02	K01468	imidazolonepropionase
PP_5031	131.98	2.16	1.98E-02	K16234	amino acid ABC transporter permease
PP_5350	882.29	2.29	2.00E-02	K19337	RpiR family transcriptional regulator
PP_0460	3260.53	2.27	2.01E-02	K02982	30S ribosomal protein S3
PP_5249	67.33	2.29	2.01E-02		LysR family transcriptional regulator
PP_0284	404.24	7.86	2.03E-02	K11735	GABA permease
PP_0491	575.49	2.14	2.03E-02	K00127	formate dehydrogenase subunit gamma
PP_3725	26.20	3.49	2.05E-02		acyl-CoA dehydrogenase
PP_1986	320.88	2.97	2.09E-02	K01704	isopropylmalate isomerase small subunit
PP_1726	2561.79	3.11	2.11E-02	K02055	ABC transporter substrate-binding protein
PP_3783	24.36	2.21	2.11E-02	K15650	hypothetical protein PP_3783
PP_4548	284.99	2.46	2.11E-02	K09471	FAD dependent oxidoreductase
PP_0019	2033.40	2.06	2.13E-02		hypothetical protein PP_0019
PP_0969	272.40	2.15	2.14E-02		GntR family transcriptional regulator
PP_2187	1165.98	2.68	2.14E-02		universal stress protein
PP_1000	2265.38	5.92	2.18E-02	K00611	ornithine carbamoyltransferase
PP_0898	3.19	2.30	2.19E-02		hypothetical protein PP_0898
PP_1383	3034.77	2.27	2.22E-02		BenF-like porin
PP_4453	514.04	2.10	2.24E-02	K02031	opine ABC transporter ATP-binding protein
PP_1293	67.64	2.50	2.37E-02	K03636	molybdopterin converting factor subunit 1
PP_0742	1745.69	2.01	2.37E-02	K18208	hypothetical protein PP_0742
PP_0214	1372.41	2.05	2.37E-02	K14268	4-aminobutyrate aminotransferase
PP_1522	2507.94	2.40	2.39E-02	K03704	cold shock protein CspA
PP_4925	50.95	2.20	2.39E-02		hypothetical protein PP_4925
PP_2630	19.37	2.10	2.41E-02		hypothetical protein PP_2630
PP_2054	216.29	2.57	2.43E-02		LysR family transcriptional regulator
PP_5188	288.16	2.13	2.43E-02	K05800	transcriptional regulator BkdR
PP_0466	4409.31	2.99	2.43E-02	K02931	50S ribosomal protein L5

Gene ID	baseMean (normalized)	log2 Fold Change	p- value	KO term	Description
PP_1210	1887.34	3.30	2.46E-02	K04047	DNA-binding stress protein
PP_2018	194.56	3.63	2.49E-02		BNR repeat-containing protein
PP_5338	343.79	4.20	2.49E-02	K01744	aspartate ammonia-lyase
PP_2602	132.17	2.83	2.54E-02		oxidoreductase domain-containing protein
PP_4245	797.61	2.29	2.62E-02		siderophore biosynthesis protein. partial
PP_4868	302.16	2.34	2.63E-02	K00763	nicotinate phosphoribosyltransferase
PP_0338	2100.21	4.61	2.64E-02	K00627	dihydrolipoamide acetyltransferase
PP_4413	89.38	2.14	2.64E-02		hypothetical protein PP_4413
PP_3461	44.74	2.48	2.65E-02	K06871	radical SAM domain-containing protein
PP_3915	2.66	3.02	2.67E-02		hypothetical protein PP_3915
PP_2990	1048.71	2.18	2.69E-02		MerR family transcriptional regulator
PP_1171	55.02	3.05	2.71E-02	K18981	NAD-dependent epimerase/dehydratase
PP_1247	256.62	3.89	2.72E-02		hypothetical protein PP_1247
PP_0025	41.72	2.02	2.73E-02		hypothetical protein PP_0025
PP_3185	40.88	2.00	2.74E-02		TenA family transcriptional activator
PP_3903	29.07	2.03	2.74E-02		hypothetical protein PP_3903
PP_2870	56.82	2.61	2.75E-02	K02055	PotD/PotF family extracellular solute-binding protein
PP_4727	1642.26	2.21	2.76E-02	K04043	molecular chaperone DnaK
PP_4484	321.84	2.10	2.77E-02	K10023	polar amino acid ABC transporter inner membrane subunit
PP_5140	262.07	5.44	2.78E-02		MerR family transcriptional regulator
PP_0974	97.77	3.00	2.84E-02		hypothetical protein PP_0974
PP_1149	8455.21	7.92	2.88E-02		hypothetical protein PP_1149
PP_4321	193.94	2.56	2.90E-02	K02199	thiol--disulfide oxidoreductase
PP_4340	752.48	2.88	2.92E-02	K03413	response regulator receiver protein
PP_3745	281.26	4.82	2.93E-02	K00104	glycolate oxidase subunit GlcD
PP_1060	578.36	2.16	2.95E-02		glutamate synthase. large subunit
PP_4149	183.95	2.28	2.97E-02	K13895	binding-protein-dependent transport system inner membrane protein
PP_4234	861.49	3.47	3.00E-02		(2Fe-2S)-binding protein
PP_4631	31.21	2.11	3.04E-02	K09932	hypothetical protein PP_4631
PP_0281	85.64	2.21	3.07E-02	K23056	polar amino acid ABC transporter inner membrane subunit
PP_2179	13.53	3.41	3.07E-02	K07010	peptidase C26
PP_3046	16.23	2.31	3.11E-02		hypothetical protein PP_3046
PP_0320	159.56	3.39	3.16E-02	K03406	methyl-accepting chemotaxis sensory transducer
PP_3876	2.07	9.02	3.19E-02		hypothetical protein PP_3876
PP_0684	481.80	3.35	3.20E-02	K03773	FKBP-type peptidylprolyl isomerase
PP_4230	270.89	3.34	3.24E-02	K07141	hypothetical protein PP_4230
PP_2847	18.34	2.14	3.24E-02	K03192	HupE/UreJ protein
PP_4393	3128.46	2.19	3.26E-02	K03415	chemotaxis protein CheV
PP_0896	88.94	3.05	3.31E-02		nitrilase/cyanide hydratase and apolipoprotein N-acyltransferase
PP_4356	83.92	2.17	3.33E-02	K02418	flagellar assembly protein FliO
PP_3887	29.93	3.64	3.36E-02		hypothetical protein PP_3887
PP_1791	2741.45	2.19	3.38E-02	K01666	aldolase
PP_3462	151.32	2.69	3.38E-02		hypothetical protein PP_3462
PP_5085	2521.94	3.64	3.40E-02	K00029	malic enzyme
PP_3230	55.40	2.52	3.47E-02	K07100	phosphoribosyl transferase domain-containing protein
PP_5178	1320.56	2.16	3.49E-02	K11075	binding-protein-dependent transport system inner membrane protein
PP_3572	18.15	2.29	3.52E-02		hypothetical protein PP_3572
PP_2655	54.38	4.44	3.53E-02		hypothetical protein PP_2655
PP_0360	49.01	3.27	3.70E-02	K02019	ModE family transcriptional regulator
PP_0993	36.34	4.51	3.74E-02		hypothetical protein PP_0993
PP_5255	69.88	2.43	3.75E-02		isochorismatase superfamily hydrolase
PP_4662	56.79	3.25	3.82E-02		antibiotic biosynthesis monooxygenase
PP_5200	791.12	2.20	3.90E-02	K01262	peptidase M24
PP_4093	158.09	6.53	3.91E-02		ISPpu15. transposase Orf1
PP_0162	14.66	2.15	3.93E-02		ECF subfamily RNA polymerase sigma factor
PP_3278	14.34	2.11	3.98E-02	K02609	phenylacetate-CoA oxygenase subunit PaaA
PP_0464	2429.73	3.55	4.00E-02	K02874	50S ribosomal protein L14
PP_5209	1584.22	3.69	4.01E-02	K02415	flagellar basal body protein FliL
PP_4318	16.86	2.86	4.05E-02		ISPpu8. transposase
PP_3747	83.19	3.97	4.15E-02	K11473	glycolate oxidase iron-sulfur subunit
PP_0509	266.72	2.26	4.29E-02		hypothetical protein PP_0509
PP_3276	5.19	2.95	4.35E-02	K02611	phenylacetate-CoA oxygenase subunit PaaI
PP_0056	1301.53	2.44	4.36E-02	K00108	GMC family oxidoreductase
PP_0355	718.36	2.12	4.36E-02		response regulator receiver protein
PP_0461	2037.33	2.46	4.41E-02	K02878	50S ribosomal protein L16
PP_3893	12.29	2.34	4.42E-02	K02314	phage DNA helicase
PP_4238	5.33	2.42	4.43E-02		hypothetical protein PP_4238
PP_1786	5724.83	2.33	4.44E-02		glycosyl transferase family protein
PP_0250	771.75	3.61	4.44E-02	K04762	RNA-binding S4 domain-containing protein
PP_4185	18139.28	2.90	4.46E-02	K01902	succinyl-CoA synthetase subunit alpha
PP_2674	84.82	7.04	4.46E-02	K00114	quinoprotein ethanol dehydrogenase
PP_3779	575.59	2.27	4.53E-02		LysR family transcriptional regulator
PP_2637	19.19	2.25	4.63E-02	K20542	endo-1.4-D-glucanase
PP_2846	16.84	2.19	4.66E-02	K03187	urease accessory protein UreE
PP_1249	43.05	3.13	4.67E-02		hypothetical protein PP_1249
PP_0016	353.46	2.29	4.68E-02		hypothetical protein PP_0016
PP_0727	76.06	2.07	4.76E-02	K23150	hypothetical protein PP_0727
PP_4331	439.45	2.62	4.77E-02		hypothetical protein PP_4331
PP_4871	425.15	2.26	4.80E-02		hypothetical protein PP_4871
PP_3748	11.17	6.73	4.81E-02	K11477	hypothetical protein PP_3748

Supplementary Table 5. Gene assignment to groups.

Group	Gene ID	KO	Description
**Ribosomal protein**	PP_0009	K02914	50S ribosomal protein L34
**Ribosomal protein**	PP_0389	K02970	30S ribosomal protein S21
**Ribosomal protein**	PP_0443	K02867	50S ribosomal protein L11
**Ribosomal protein**	PP_0444	K02863	50S ribosomal protein L1
**Ribosomal protein**	PP_0445	K02864	50S ribosomal protein L10
**Ribosomal protein**	PP_0446	K02935	50S ribosomal protein L7/L12
**Ribosomal protein**	PP_0449	K02950	30S ribosomal protein S12
**Ribosomal protein**	PP_0450	K02992	30S ribosomal protein S7
**Ribosomal protein**	PP_0453	K02946	30S ribosomal protein S10
**Ribosomal protein**	PP_0454	K02906	50S ribosomal protein L3
**Ribosomal protein**	PP_0455	K02926	50S ribosomal protein L4
**Ribosomal protein**	PP_0456	K02892	50S ribosomal protein L23
**Ribosomal protein**	PP_0457	K02886	50S ribosomal protein L2
**Ribosomal protein**	PP_0458	K02965	30S ribosomal protein S19
**Ribosomal protein**	PP_0459	K02890	50S ribosomal protein L22
**Ribosomal protein**	PP_0460	K02982	30S ribosomal protein S3
**Ribosomal protein**	PP_0461	K02878	50S ribosomal protein L16
**Ribosomal protein**	PP_0462	K02904	50S ribosomal protein L29
**Ribosomal protein**	PP_0463	K02961	30S ribosomal protein S17
**Ribosomal protein**	PP_0464	K02874	50S ribosomal protein L14
**Ribosomal protein**	PP_0465	K02895	50S ribosomal protein L24
**Ribosomal protein**	PP_0466	K02931	50S ribosomal protein L5
**Ribosomal protein**	PP_0467	K02954	30S ribosomal protein S14
**Ribosomal protein**	PP_0468	K02994	30S ribosomal protein S8
**Ribosomal protein**	PP_0469	K02933	50S ribosomal protein L6
**Ribosomal protein**	PP_0470	K02881	50S ribosomal protein L18
**Ribosomal protein**	PP_0471	K02988	30S ribosomal protein S5
**Ribosomal protein**	PP_0472	K02907	50S ribosomal protein L30
**Ribosomal protein**	PP_0473	K02876	50S ribosomal protein L15
**Ribosomal protein**	PP_0475	K02919	50S ribosomal protein L36
**Ribosomal protein**	PP_0476	K02952	30S ribosomal protein S13
**Ribosomal protein**	PP_0477	K02948	30S ribosomal protein S11
**Ribosomal protein**	PP_0478	K02986	30S ribosomal protein S4
**Ribosomal protein**	PP_0480	K02879	50S ribosomal protein L17
**Ribosomal protein**	PP_0600	K02968	30S ribosomal protein S20
**Ribosomal protein**	PP_0688	K02888	50S ribosomal protein L21
**Ribosomal protein**	PP_0689	K02899	50S ribosomal protein L27
**Ribosomal protein**	PP_0721	K02897	50S ribosomal protein L25
**Ribosomal protein**	PP_0951	K05808	sigma 54 modulation protein/ribosomal protein S30EA
**Ribosomal protein**	PP_1197	K14441	30S ribosomal protein S12 methylthiotransferase
**Ribosomal protein**	PP_1315	K02871	50S ribosomal protein L13
**Ribosomal protein**	PP_1316	K02996	30S ribosomal protein S9
**Ribosomal protein**	PP_1462	K02959	30S ribosomal protein S16
**Ribosomal protein**	PP_1465	K02884	50S ribosomal protein L19
**Ribosomal protein**	PP_1591	K02967	30S ribosomal protein S2
**Ribosomal protein**	PP_1772	K02945	30S ribosomal protein S1
**Ribosomal protein**	PP_1911	K02911	50S ribosomal protein L32
**Ribosomal protein**	PP_2467	K02916	50S ribosomal protein L35
**Ribosomal protein**	PP_2468	K02887	50S ribosomal protein L20
**Ribosomal protein**	PP_2956		ribosomal protein S6 modification protein-like protein
**Ribosomal protein**	PP_4709	K02956	30S ribosomal protein S15
**Ribosomal protein**	PP_4818	K02687	50S ribosomal protein L11 methyltransferase
**Ribosomal protein**	PP_4874	K02939	50S ribosomal protein L9
**Ribosomal protein**	PP_4876	K02963	30S ribosomal protein S18
**Ribosomal protein**	PP_4877	K02990	30S ribosomal protein S6
**Ribosomal protein**	PP_5087	K02909	50S ribosomal protein L31
**Ribosomal protein**	PP_5281	K02913	50S ribosomal protein L33
**Ribosomal protein**	PP_5282	K02902	50S ribosomal protein L28
**sigma factor**	PP_0161		FecR anti-FecI sigma factor
**sigma factor**	PP_0162		ECF subfamily RNA polymerase sigma factor
**sigma factor**	PP_0191	K07740	anti-RNA polymerase sigma 70 factor
**sigma factor**	PP_0351	K07165	FecR anti-FecI sigma factor
**sigma factor**	PP_0352	K23514	RNA polymerase sigma factor
**sigma factor**	PP_0387	K03086	RNA polymerase sigma factor RpoD

**sigma factor**	PP_0667		ECF subfamily RNA polymerase sigma factor
**sigma factor**	PP_0700		FecR anti-FecI sigma factor
**sigma factor**	PP_0703	K07165	FecR anti-FecI sigma factor
**sigma factor**	PP_0704		ECF subfamily RNA polymerase sigma factor
**sigma factor**	PP_0865		ECF subfamily RNA polymerase sigma-24 subunit
**sigma factor**	PP_0866	K07165	FecR anti-FecI sigma factor
**sigma factor**	PP_0951	K05808	sigma 54 modulation protein/ribosomal protein S30EA
**sigma factor**	PP_0952	K03092	RNA polymerase factor sigma-54
**sigma factor**	PP_0994	K03088	RNA polymerase sigma factor
**sigma factor**	PP_1007		FecR anti-FecI sigma factor
**sigma factor**	PP_1008		ECF subfamily RNA polymerase sigma factor
**sigma factor**	PP_1427	K03088	RNA polymerase sigma factor AlgU
**sigma factor**	PP_1428	K03597	anti sigma-E protein. RseA
**sigma factor**	PP_1429	K03598	sigma E regulatory protein MucB/RseB
**sigma factor**	PP_1623	K03087	RNA polymerase sigma factor RpoS
**sigma factor**	PP_2088	K03088	RNA polymerase sigma factor SigX
**sigma factor**	PP_2166		anti-sigma-factor antagonist
**sigma factor**	PP_2192		ECF subfamily RNA polymerase sigma factor
**sigma factor**	PP_2888	K03088	ECF subfamily RNA polymerase sigma-24 subunit
**sigma factor**	PP_2889		transmembrane anti-sigma factor
**sigma factor**	PP_3006	K03088	RNA polymerase sigma factor
**sigma factor**	PP_3086		RNA polymerase sigma-70 factor
**sigma factor**	PP_3555		FecR anti-FecI sigma factor
**sigma factor**	PP_3576	K07165	FecR anti-FecI sigma factor
**sigma factor**	PP_3577		ECF subfamily RNA polymerase sigma factor
**sigma factor**	PP_4208		RNA polymerase sigma factor
**sigma factor**	PP_4244		extracytoplasmic-function sigma-70 factor
**sigma factor**	PP_4341	K02405	flagellar biosynthesis sigma factor
**sigma factor**	PP_4364	K20978	anti-sigma-factor antagonist
**sigma factor**	PP_4395	K02398	anti-sigma-28 factor FlgM
**sigma factor**	PP_4553	K03088	ECF subfamily RNA polymerase sigma factor
**sigma factor**	PP_4607	K07165	FecR anti-FecI sigma factor
**sigma factor**	PP_4608		ECF subfamily RNA polymerase sigma factor
**sigma factor**	PP_4611	K23514	RNA polymerase sigma factor
**sigma factor**	PP_5108	K03089	RNA polymerase factor sigma-32
**Chaperones**	PP_0407	K05801	molecular chaperone DnaJ
**Chaperones**	PP_0724	K02494	molecular chaperone LolB
**Chaperones**	PP_0845	K04082	co-chaperone HscB
**Chaperones**	PP_0846	K04044	chaperone protein HscA
**Chaperones**	PP_1361	K04077	molecular chaperone GroEL
**Chaperones**	PP_1600	K06142	outer membrane chaperone Skp
**Chaperones**	PP_1890	K07346	pili assembly chaperone
**Chaperones**	PP_2361	K07346	type 1 pili usher pathway chaperone CsuC
**Chaperones**	PP_3095	K11907	chaperone-associated ATPase
**Chaperones**	PP_3316	K03696	chaperone-associated ATPase
**Chaperones**	PP_3970		molecular chaperone GroES
**Chaperones**	PP_4365	K02413	flagellar biosynthesis chaperone
**Chaperones**	PP_4726	K03686	molecular chaperone DnaJ
**Chaperones**	PP_4727	K04043	molecular chaperone DnaK
**Chaperones**	PP_4849	K04046	molecular chaperone DnaK
**RNA polymerases**	PP_0162		ECF subfamily RNA polymerase sigma factor
**RNA polymerases**	PP_0191	K07740	anti-RNA polymerase sigma 70 factor
**RNA polymerases**	PP_0352	K23514	RNA polymerase sigma factor
**RNA polymerases**	PP_0387	K03086	RNA polymerase sigma factor RpoD
**RNA polymerases**	PP_0447	K03043	DNA-directed RNA polymerase subunit beta
**RNA polymerases**	PP_0448	K03046	DNA-directed RNA polymerase subunit beta\
**RNA polymerases**	PP_0479	K03040	DNA-directed RNA polymerase subunit alpha
**RNA polymerases**	PP_0667		ECF subfamily RNA polymerase sigma factor
**RNA polymerases**	PP_0704		ECF subfamily RNA polymerase sigma factor
**RNA polymerases**	PP_0865		ECF subfamily RNA polymerase sigma-24 subunit
**RNA polymerases**	PP_0952	K03092	RNA polymerase factor sigma-54
**RNA polymerases**	PP_0994	K03088	RNA polymerase sigma factor
**RNA polymerases**	PP_1008		ECF subfamily RNA polymerase sigma factor
**RNA polymerases**	PP_1427	K03088	RNA polymerase sigma factor AlgU

**RNA polymerases**	PP_1623	K03087	RNA polymerase sigma factor RpoS
**RNA polymerases**	PP_2088	K03088	RNA polymerase sigma factor SigX
**RNA polymerases**	PP_2192		ECF subfamily RNA polymerase sigma factor
**RNA polymerases**	PP_2266		DNA-directed RNA polymerase. bacteriophage and mitochondrial type
**RNA polymerases**	PP_2888	K03088	ECF subfamily RNA polymerase sigma-24 subunit
**RNA polymerases**	PP_3006	K03088	RNA polymerase sigma factor
**RNA polymerases**	PP_3086		RNA polymerase sigma-70 factor
**RNA polymerases**	PP_3577		ECF subfamily RNA polymerase sigma factor
**RNA polymerases**	PP_4208		RNA polymerase sigma factor
**RNA polymerases**	PP_4553	K03088	ECF subfamily RNA polymerase sigma factor
**RNA polymerases**	PP_4608		ECF subfamily RNA polymerase sigma factor
**RNA polymerases**	PP_4611	K23514	RNA polymerase sigma factor
**RNA polymerases**	PP_5108	K03089	RNA polymerase factor sigma-32
**RNA polymerases**	PP_5301	K03060	DNA-directed RNA polymerase subunit omega
**stress**	PP_1210	K04047	DNA-binding stress protein
**stress**	PP_1269		universal stress protein
**stress**	PP_2132	K06149	universal stress protein
**stress**	PP_2187		universal stress protein
**stress**	PP_2326		universal stress protein
**stress**	PP_2648		universal stress protein
**stress**	PP_2745		universal stress protein
**stress**	PP_3156		universal stress protein
**stress**	PP_3237		universal stress protein
**stress**	PP_3288		universal stress protein
**stress**	PP_3290		universal stress protein
**stress**	PP_3294		universal stress protein
**oxidative stress**	PP_0115	K03781	hydroperoxidase II
**oxidative stress**	PP_0235	K24158	peroxidase
**oxidative stress**	PP_0777	K00432	glutathione peroxidase
**oxidative stress**	PP_1686	K00432	glutathione peroxidase
**oxidative stress**	PP_1874	K00432	glutathione peroxidase
**oxidative stress**	PP_2422		alkylhydroperoxidase
**oxidative stress**	PP_2439	K24119	alkyl hydroperoxide reductase
**oxidative stress**	PP_2561		heme peroxidase
**oxidative stress**	PP_2943	K00428	cytochrome c551 peroxidase
**oxidative stress**	PP_2959		alkylhydroperoxidase
**oxidative stress**	PP_3248	K07223	Dyp-type peroxidase
**oxidative stress**	PP_3639		alkylhydroperoxidase
**oxidative stress**	PP_3668	K03782	catalase/peroxidase HPI
**oxidative stress**	PP_0481	K03781	catalase
**oxidative stress**	PP_2887	K03781	catalase domain-containing protein
**oxidative stress**	PP_0915	K04564	superoxide dismutase
**oxidative stress**	PP_0946	K04564	superoxide dismutase
**Flagella**	PP_4335	K02557	flagellar motor protein MotD
**Flagella**	PP_4336	K02556	flagellar motor protein
**Flagella**	PP_4341	K02405	flagellar biosynthesis sigma factor
**Flagella**	PP_4342	K04562	flagellar number regulator FleN
**Flagella**	PP_4343	K02404	flagellar biosynthesis regulator FlhF
**Flagella**	PP_4344	K02400	flagellar biosynthesis protein FlhA
**Flagella**	PP_4352	K02401	flagellar biosynthesis protein FlhB
**Flagella**	PP_4353	K02421	flagellar biosynthesis protein FliR
**Flagella**	PP_4354	K02420	flagellar biosynthesis protein FliQ
**Flagella**	PP_4355	K02419	flagellar biosynthesis protein FliP
**Flagella**	PP_4356	K02418	flagellar assembly protein FliO
**Flagella**	PP_4357	K02417	flagellar motor switch protein
**Flagella**	PP_4358	K02416	flagellar motor switch protein FliM
**Flagella**	PP_4359	K02415	flagellar basal body protein FliL
**Flagella**	PP_4361	K02414	flagellar hook-length control protein
**Flagella**	PP_4365	K02413	flagellar biosynthesis chaperone
**Flagella**	PP_4366	K02412	flagellum-specific ATP synthase
**Flagella**	PP_4367	K02411	flagellar assembly protein H
**Flagella**	PP_4368	K02410	flagellar motor switch protein G
**Flagella**	PP_4369	K02409	flagellar MS-ring protein

**Flagella**	PP_4370	K02408	flagellar hook-basal body protein FliE
**Flagella**	PP_4375	K02422	flagellar protein FliS
**Flagella**	PP_4376	K02407	flagellar cap protein FliD
**Flagella**	PP_4377	K06603	flagellin FlaG
**Flagella**	PP_4378	K02406	flagellin FliC
**Flagella**	PP_4380	K02397	flagellar hook-associated protein FlgL
**Flagella**	PP_4381	K02396	flagellar hook-associated protein FlgK
**Flagella**	PP_4382	K02395	flagellar rod assembly protein FlgJ
**Flagella**	PP_4383	K02394	flagellar basal body P-ring biosynthesis protein FlgA
**Flagella**	PP_4384	K02393	flagellar basal body L-ring protein
**Flagella**	PP_4385	K02392	flagellar basal body rod protein FlgG
**Flagella**	PP_4386	K02391	flagellar basal body rod protein FlgF
**Flagella**	PP_4388	K02390	flagellar hook protein FlgE
**Flagella**	PP_4389	K02389	flagellar basal body rod modification protein
**Flagella**	PP_4390	K02388	flagellar basal body rod protein FlgC
**Flagella**	PP_4391	K02387	flagellar basal-body rod protein FlgB
**Flagella**	PP_4394	K02386	flagellar basal body P-ring biosynthesis protein FlgA
**Flagella**	PP_4904	K02557	flagellar motor protein MotB
**Flagella**	PP_4905	K02556	flagellar motor protein MotA
**Flagella**	PP_5209	K02415	flagellar basal body protein FliL
**Cytochromes**	PP_0103	K02275	cytochrome c oxidase subunit II
**Cytochromes**	PP_0104	K02274	cytochrome c oxidase subunit I
**Cytochromes**	PP_0105	K02258	cytochrome C oxidase assembly protein
**Cytochromes**	PP_0106	K02276	cytochrome c oxidase subunit III
**Cytochromes**	PP_0109	K02259	cytochrome oxidase assembly
**Cytochromes**	PP_0125		cytochrome c-type protein
**Cytochromes**	PP_0126		cytochrome c4
**Cytochromes**	PP_0180	K07243	cytochrome c family protein
**Cytochromes**	PP_0813	K02298	cytochrome o ubiquinol oxidase subunit I
**Cytochromes**	PP_0814	K02299	cytochrome o ubiquinol oxidase subunit III
**Cytochromes**	PP_0815	K02300	cytochrome o ubiquinol oxidase. protein CyoD
**Cytochromes**	PP_1317	K00411	ubiquinol-cytochrome c reductase. iron-sulfur subunit
**Cytochromes**	PP_1318	K00412	ubiquinol--cytochrome c reductase. cytochrome b
**Cytochromes**	PP_1319	K00413	ubiquinol--cytochrome c reductase. cytochrome c1
**Cytochromes**	PP_1460		cytochrome c assembly protein
**Cytochromes**	PP_1841		cytochrome c family protein
**Cytochromes**	PP_1955		cytochrome P450 family protein
**Cytochromes**	PP_2010	K12262	cytochrome B561
**Cytochromes**	PP_2479		cytochrome c family protein
**Cytochromes**	PP_2675		cytochrome c-type protein
**Cytochromes**	PP_2886		cytochrome B561
**Cytochromes**	PP_2943	K00428	cytochrome c551 peroxidase
**Cytochromes**	PP_3332		cytochrome c-type protein
**Cytochromes**	PP_3486		cytochrome c
**Cytochromes**	PP_3822	K19713	cytochrome c family protein
**Cytochromes**	PP_3823		cytochrome c-type protein
**Cytochromes**	PP_4193	K00241	succinate dehydrogenase. cytochrome b556 subunit
**Cytochromes**	PP_4250	K00404	cbb3-type cytochrome c oxidase subunit I
**Cytochromes**	PP_4251	K00405	cbb3-type cytochrome c oxidase subunit II
**Cytochromes**	PP_4252	K00407	cytochrome c oxidase. cbb3-type. CcoQ subunit
**Cytochromes**	PP_4253	K00406	cytochrome c oxidase. cbb3-type subunit III
**Cytochromes**	PP_4255	K00404	cbb3-type cytochrome c oxidase subunit I
**Cytochromes**	PP_4256	K00405	cbb3-type cytochrome c oxidase subunit II
**Cytochromes**	PP_4257	K00407	cbb3-type cytochrome oxidase subunit
**Cytochromes**	PP_4258	K00406	cytochrome c oxidase. cbb3-type subunit III
**Cytochromes**	PP_4262		cbb3-type cytochrome oxidase maturation protein
**Cytochromes**	PP_4322	K02198	cytochrome C biogenesis protein CcmF
**Cytochromes**	PP_4323	K02197	cytochrome C biogenesis protein CcmE
**Cytochromes**	PP_4324	K02196	cytochrome C biogenesis protein CcmD
**Cytochromes**	PP_4327	K02193	cytochrome c biogenesis protein CcmA
**Cytochromes**	PP_4650	K00426	cytochrome d ubiquinol oxidase subunit II
**Cytochromes**	PP_4970		cytochrome c\
**Cytochromes**	PP_4982		cytochrome B561
**Cytochromes**	PP_5267		cytochrome c5
**Cytochromes**	PP_5378		cytochrome c family protein

Supplementary Table 6. Transcripts driving the principal components separation between exponential and stationary phases

Gene_ID	PC	Product_description	Gene_Name
PP_1786	PC1	glycosyl transferase	PP_1786
PP_1222	PC1	Tol biopolymer transport system protein	*tolB*
PP_1464	PC1	tRNA (guanosine(37)-N1)-methyltransferase TrmD	*trmD*
PP_5417	PC1	ATP synthase subunit b	*atpF*
PP_0450	PC1	30S ribosomal protein S7	*rpsG*
PP_5044	PC1	ribosome associated GTPase	*typA*
PP_0339	PC1	pyruvate dehydrogenase E1 component	*aceE*
PP_0858	PC1	methionine/glutamine aminotransferase	PP_0858
PP_5419	PC1	ATP synthase subunit a	*atpB*
PP_5415	PC1	ATP synthase subunit alpha	*atpA*
PP_0478	PC1	30S ribosomal protein S4	*rpsD*
PP_0455	PC1	50S ribosomal protein L4	*rplD*
PP_0815	PC1	cytochrome bo terminal oxidase subunit IV	*cyoD*
PP_0451	PC1	elongation factor G 1	*fusA*
PP_1791	PC1	aldolase/synthase	PP_1791
PP_4009	PC2	ATP-dependent Clp protease adapter protein ClpS	*clpS*
PP_2343	PC2	hypothetical protein	PP_2343
PP_0951	PC2	ribosome hibernation promoting factor	*hpf*
PP_0950	PC2	phosphotransferase system subunit IIA	*ptsN*
PP_4470	PC2	Arc domain-containing transcriptional regulator	PP_4470
PP_2471	PC2	integration host factor subunit alpha	*ihfA*
PP_4204	PC2	type II toxin-antitoxin MqsRA system antitoxin/DNA-binding transcriptional repressor	*mqsA*
PP_4205	PC2	mRNA interferase MqsR	*mqsR*
PP_3832	PC2	RsmE	*rsmE*
PP_5558	PC2	Xre family transcriptional regulator	PP_5558
PP_5234	PC2	NRII(GlnL/NtrB) phosphatase activator	*glnK*
PP_0191	PC2	transcriptional regulator AlgQ	*algQ*
PP_1305	PC2	colicin-E7 immunity protein	*imm*
PP_5119	PC2	TetR family transcriptional regulator	PP_5119
PP_5693	PC2	NA	NA
PP_1792	PC1	NA	NA
PP_0173	PC1	NA	NA
PP_5550	PC2	NA	NA
PP_1790	PC1	NA	NA
PP_0961	PC2	NA	NA
PP_1788	PC1	NA	NA
PP_3704	PC2	NA	NA
PP_5146	PC2	NA	NA
PP_1789	PC1	NA	NA
PP_5629	PC2	NA	NA

Supplementary Table 7. Genes with expression significantly deviating from the wild-type-based prediction in pairwise comparisons in stationary phase under mixed substrate conditions.

Comparison	Gene ID	WT mean	Mutant mean	Log2 fold change	residuals	fitted	adjusted p-value	Description	Gene Name
7260 vs 7301	PP_1947	3.50	1.16	-7.78	-6.25	3.02	2.47E-06	NA	NA
7260 vs 7301	PP_1950	3.62	1.38	-7.44	-5.80	3.11	1.97E-05	hypothetical protein	PP_1950
7260 vs 7301	PP_1948	3.59	1.52	-6.86	-5.24	3.08	2.35E-04	benzaldehyde dehydrogenase	PP_1948
7260 vs 7301	PP_3162	3.14	1.18	-6.48	-5.25	2.75	2.35E-04	benzoate 1 2-dioxygenase subunit beta	*benB*
7260 vs 7301	PP_3167	2.67	0.88	-5.96	-5.12	2.40	3.63E-04	benzoate transport protein	*benE-II*
7260 vs 7301	PP_3161	3.63	1.60	-6.73	-5.08	3.12	3.78E-04	benzoate 1 2-dioxygenase subunit alpha	*benA*
7260 vs 7301	PP_1949	3.59	1.61	-6.57	-4.95	3.09	5.42E-04	GMC family oxidoreductase	PP_1949
7260 vs 7301	PP_2733	2.57	0.85	-5.71	-4.95	2.33	5.42E-04	NA	NA
7260 vs 7301	PP_1946	3.66	1.68	-6.57	-4.89	3.14	6.48E-04	NA	NA
7260 vs 7301	PP_3164	2.79	1.07	-5.73	-4.78	2.49	1.02E-03	NA	NA
7260 vs 7301	PP_3165	3.16	1.38	-5.92	-4.66	2.77	1.66E-03	benzoate MFS transporter	*benK*
7260 vs 7301	PP_3166	3.02	1.28	-5.77	-4.63	2.66	1.66E-03	catechol 1 2-dioxygenase	*catA-II*
7260 vs 7301	PP_1951	3.28	1.48	-5.99	-4.63	2.86	1.66E-03	NA	NA
7260 vs 7301	PP_3163	2.84	1.16	-5.56	-4.57	2.53	1.99E-03	benzoate 1 2-dioxygenase electron transfer component	*benC*
7260 vs 7301	PP_1944	4.10	2.19	-6.36	-4.30	3.47	6.70E-03	aminomethyltransferase	PP_1944
7260 vs 7301	PP_3168	2.80	1.23	-5.22	-4.25	2.50	7.68E-03	porin-like protein	*nicP-I*
7260 vs 7301	PP_3312	2.25	3.35	3.67	4.23	2.09	7.94E-03	NA	NA
7260 vs 7301	PP_0201	2.67	3.64	3.21	4.13	2.40	1.16E-02	hypothetical protein	PP_0201
7260 vs 7301	PP_5428	1.84	3.01	3.90	4.11	1.78	1.18E-02	NA	NA
7260 vs 7301	PP_3089	3.03	1.47	-5.19	-4.03	2.67	1.61E-02	NA	NA
7260 vs 7301	PP_2889	1.12	2.43	4.36	3.96	1.25	2.06E-02	NA	NA
7260 vs 7301	PP_0136	2.00	3.05	3.48	3.83	1.91	3.15E-02	hypothetical protein	PP_0136
7260 vs 7301	PP_1489	1.32	0.25	-3.54	-3.83	1.40	3.15E-02	CheW domain-containing protein	PP_1489
7260 vs 7301	PP_1952	2.99	1.50	-4.95	-3.82	2.64	3.15E-02	NA	NA
7260 vs 7301	orf8052	3.05	1.55	-4.98	-3.80	2.69	3.28E-02	NA	NA
7260 vs 7301	PP_4490	1.88	2.95	3.53	3.78	1.82	3.46E-02	phenylalanine-4-hydroxylase	*phhA*
7260 vs 7301	clcD	3.16	1.66	-4.99	-3.72	2.77	4.28E-02	NA	NA
7260 vs 7301	PP_2513	2.50	1.17	-4.40	-3.68	2.27	4.68E-02	NA	NA
7260 vs 7262	clcA	3.55	0.45	-10.30	-8.06	2.77	5.22E-12	NA	NA
7260 vs 7262	clcD	3.16	0.34	-9.37	-7.67	2.54	5.71E-11	NA	NA
7260 vs 7262	clcB	3.42	0.55	-9.54	-7.45	2.69	2.00E-10	NA	NA
7260 vs 7262	orf10938	3.32	0.65	-8.90	-6.92	2.63	7.08E-09	NA	NA
7260 vs 7262	clcE	3.52	0.85	-8.87	-6.61	2.74	4.84E-08	NA	NA
7260 vs 7262	orf94175	2.63	0.78	-6.12	-5.03	2.23	4.69E-04	NA	NA
7260 vs 7262	PP_3403	1.66	2.97	4.33	4.46	1.68	6.91E-03	NA	NA
7260 vs 7262	orf58432	2.97	1.19	-5.91	-4.30	2.43	1.24E-02	NA	NA
7260 vs 7262	clcR	1.93	0.60	-4.41	-4.27	1.83	1.25E-02	NA	NA
7260 vs 7262	PP_2951	2.01	3.05	3.45	4.05	1.88	2.57E-02	TetR family transcriptional regulator	PP_2951
7260 vs 7262	orf73676	2.21	0.83	-4.57	-4.03	1.99	2.57E-02	NA	NA
7260 vs 7262	orf84338	2.38	0.93	-4.83	-4.04	2.09	2.57E-02	NA	NA
7260 vs 7262	orf85934	2.91	1.24	-5.55	-4.02	2.39	2.57E-02	NA	NA
7260 vs 7262	PP_4433	0.99	2.44	4.81	3.97	1.30	2.63E-02	amino acid MFS transporter	PP_4433
7260 vs 7262	orf73029	1.92	0.69	-4.10	-3.97	1.83	2.63E-02	NA	NA
7260 vs 7262	orf89247	2.58	1.06	-5.06	-3.99	2.21	2.63E-02	NA	NA
7260 vs 7262	PP_2501	2.13	3.08	3.16	3.93	1.95	2.73E-02	integrase	PP_2501
7260 vs 7262	PP_2981	2.11	3.07	3.20	3.94	1.94	2.73E-02	cointegrate resolution protein S	*tnpS*
7260 vs 7262	orf94689	2.06	0.80	-4.17	-3.83	1.91	3.81E-02	NA	NA
7260 vs 7262	PP_2949	1.11	2.46	4.48	3.79	1.36	3.92E-02	permease	PP_2949
7260 vs 7262	PP_4064	2.12	3.04	3.05	3.79	1.94	3.92E-02	isovaleryl-CoA dehydrogenase	ivd
7260 vs 7262	orf95213	2.50	1.07	-4.77	-3.80	2.16	3.92E-02	NA	NA
7260 vs 7262	orf100952	2.02	0.80	-4.05	-3.77	1.89	4.01E-02	NA	NA
7260 vs 7262	orf80480	2.62	1.15	-4.90	-3.76	2.23	4.01E-02	NA	NA

7260 vs 7262	orf80908	2.48	1.07	-4.69	-3.75	2.15	4.02E-02	NA	NA
7260 vs 7262	PP_3089	3.03	1.39	-5.46	-3.73	2.47	4.14E-02	NA	NA
7260 vs 7262	orf97571	1.94	0.77	-3.89	-3.71	1.84	4.42E-02	NA	NA
7260 vs 7262	PP_3399	2.01	2.94	3.10	3.68	1.88	4.47E-02	minor curlin subunit CsgB	PP_3399
7260 vs 7262	PP_3495	2.72	3.35	2.09	3.68	2.29	4.47E-02	tRNA (adenine(22)-N(1))-methyltransferase	*trmK*
7260 vs 7262	orf79976	2.31	0.99	-4.38	-3.68	2.05	4.47E-02	NA	NA
7260 vs 7262	PP_5393	1.86	2.85	3.29	3.66	1.79	4.51E-02	metal-binding chaperone	PP_5393
7260 vs 7262	PP_3297	1.40	2.58	3.95	3.66	1.53	4.51E-02	NA	NA
7260 vs 7262	PP_5598	1.85	0.74	-3.71	-3.65	1.79	4.51E-02	NA	NA
7260 vs 7434	PP_3162	3.14	0.00	-10.42	-7.96	2.85	1.18E-11	benzoate 1 2-dioxygenase subunit beta	*benB*
7261 vs 7434	PP_3164	2.79	0.00	-9.28	-7.16	2.57	2.51E-09	NA	NA
7262 vs 7434	PP_3163	2.84	0.50	-7.75	-5.85	2.61	9.64E-06	benzoate 1 2-dioxygenase electron transfer component	*benC*
7263 vs 7434	PP_3161	3.63	1.31	-7.69	-5.41	3.26	9.49E-05	benzoate 1 2-dioxygenase subunit alpha	*benA*
7264 vs 7434	PP_3089	3.03	0.87	-7.21	-5.30	2.77	1.40E-04	NA	NA
7265 vs 7434	orf58432	2.97	0.89	-6.91	-5.08	2.72	3.77E-04	NA	NA
7266 vs 7434	PP_5465	1.74	0.00	-5.77	-4.75	1.71	1.73E-03	NA	NA
7267 vs 7434	orf97571	1.94	0.24	-5.67	-4.55	1.88	3.80E-03	NA	NA
7268 vs 7434	PP_2909	1.21	2.89	5.58	4.48	1.28	4.31E-03	carbon starvation induced protein	*csiD*
7269 vs 7434	PP_3495	2.72	4.13	4.68	4.49	2.51	4.31E-03	tRNA (adenine(22)-N(1))-methyltransferase	*trmK*
7270 vs 7434	PP_2222	2.38	3.72	4.46	4.13	2.23	1.86E-02	NA	NA
7271 vs 7434	PP_3494	2.74	3.99	4.15	4.06	2.53	2.20E-02	NA	NA
7272 vs 7434	PP_4557	2.54	3.83	4.28	4.07	2.37	2.20E-02	NA	NA
7273 vs 7434	orf71723	1.42	0.00	-4.71	-4.01	1.45	2.45E-02	NA	NA
7274 vs 7434	orf68987	2.22	0.68	-5.13	-3.96	2.11	2.83E-02	NA	NA
7275 vs 7434	PP_3623	3.06	1.40	-5.52	-3.87	2.79	3.77E-02	alcohol dehydrogenase cytochrome c subunit	*adhB*
7276 vs 7434	orf94175	2.63	1.05	-5.26	-3.86	2.44	3.77E-02	NA	NA
7277 vs 7434	PP_4858	3.11	4.21	3.65	3.83	2.84	4.15E-02	NA	NA

Supplementary Table 8. Oligonucleotides used in this study

Collection number	Primer name	Description	Sequence 5'-3'
191113	clcOperon_up_F	Fwd primer for amplification of *clc* operon upstream region from B13 for cloning into pEMG with In-Fusion kit.	GATAACAGGGTAATCTGCGTTGCCGGTAGTAG
191114	clcOperon_up_R	Reverse primer for amplification of *clc* operon upstream region from B13 for cloning into pEMG with In-Fusion kit.	CCGCTTTACGGCCAGGGCGTTGGCCACTCC
191115	clcOperon_dwn_F	Fwd primer for amplification of *clc* operon downstream region from B13 for cloning into pEMG with In-Fusion kit.	CTGGCCGTAAAGCGG
191116	clcOperon_dwn_R	Reverse primer for amplification of *clc* operon downstream region from B13 for cloning into pEMG with In-Fusion kit.	CATGCCTGCAGGTCGAGTCAGGAATACGCGC
210213	Fwd UP benABCD	Fwd primer for amplification of *benABCD* operon upstream region from UWC1 for cloning into pEMG with In-Fusion kit.	TAGGGATAACAGGGTAATCTGAGAAACAAATTTATTTCTACGCTCCCG
210214	Rev UP benABCD	Rev primer for amplification of *benABCD* operon upstream region from UWC1 for cloning into pEMG with In-Fusion kit.	GTGTACATCAAGGGTTCGCATGGCCAGGGTCTCCCTTGTTAT
210215	Fwd DWN benABCD	Fwd primer for amplification of *benABCD* operon downstream region from UWC1 for cloning into pEMG with In-Fusion kit.	ATGCGAACCCTTGATGTACACCCG
210216	Rev DWN benABCD	Rev primer for amplification of *benABCD* operon downstream region from UWC1 for cloning into pEMG with In-Fusion kit.	GCTTGCATGCCTGCAGGTCGAGCAGGCTGATCGACAGTGCGG
250408	Fwd PP_1943 BamHI	Forward primer for amplification of PP_1943 of *P. putida* ; contains a BamHI restriction site and overlaps with pUC18-miniTn7T-Lac from strain 2740.	ttcgagctcactagtggatccCTACAGTATACCGGTGAAATCAATCG
250409	Rev PP_1946 XhoI	Reverse primer for amplification of PP_1946 of *P. putida* ; contains a XhoI restriction site and overlaps with pUC18-miniTn7T-Lac from strain 2740.	accgggcccaagcttctcgagTCCATTCATAACCAGCAACGC
250105	Fwd Up HR PP_1947 EcoRI	Forward primer for amplification of the upstream flanking region of PP_1947; contains an EcoRI restriction site and overlaps with the pEMG suicide vector.	tagggataacagggtaatctGAATTC AATTATGATTTTTCCGGAAAAGTC
250106	Rev Up HR PP_1947	Reverse primer for amplification of the upstream flanking region of PP_1947.	TTGAACTCTCCATTTATTGCAG
250107	Fwd Dw HR PP_1951	Forward primer for amplification of the downstream flanking region of PP_1951; overlaps with the 3′ end of the upstream flanking region.	CCACTGCAATAAATGGAGAGTTCAACCGCTTCTCCTATTCGAAAT
250108	Rev Dw HR PP_1951 SalI	Reverse primer for amplification of the downstream flanking region of PP_1951; contains a SalI restriction site and overlaps with the pEMG suicide vector.	agaagcttgcatgcctgcagGTCGACAAGGAAAGTGGTAGCCAT
250109	Fwd Up HR PP_1952 EcoRI	Forward primer for amplification of the upstream flanking region of PP_1952; contains an EcoRI restriction site and overlaps with the pEMG suicide vector.	tagggataacagggtaatctGAATTCTTTGCTGTTCATCAATCACGGA
250110	Rev Up HR PP_1952	Reverse primer for amplification of the upstream flanking region of PP_1952.	GTGGCGAACGTCTGCT
250111	Fwd Dw HR PP_1957	Forward primer for amplification of the downstream flanking region of PP_1957; overlaps with the 3′ end of the upstream flanking region.	TGGGCTGCGAGCAGACGTTCGCCACCAAACATTTCAGGGGCAG
250112	Rev Dw HR PP_1957 SalI	Reverse primer for amplification of the downstream flanking region of PP_1957; contains a SalI restriction site and overlaps with the pEMG suicide vector.	agaagcttgcatgcctgcagGTCGACCTTCTTCACTTAAGTTGGTGC
210707	Fwd Up HR orf5994-5512 EcoRI	Forward primer for the amplification of the upstream flanking region of orf5994-5512. The primer contains a EcoRI site and overlaps with the suicide vector pEMG.	tagggataacagggtaatct GAATTC CCTACTACCGGCAACGC
210708	Rev Up HR orf5994-5512	Reverse primer for the amplification of the upstream flanking region of orf5994-5512.	CTTGGCGACCATTTCGGA
210709	Fwd Dw HR orf5994-5512	Forward primer for the amplification of the downstream flanking region of orf5994-5512. The primer overlaps with the 3′ end of the upstream flanking region.	TGTCCGAAATGGTCGCCAAG ACTCTGCTGGCCACTC
210710	Rev Dw HR orf5994-5512 SalI	Reverse primer for the amplification of the downstream flanking region of orf5994-5512. The primer contains an SalI site and overlaps with the pEMG suicide vector.	agaagcttgcatgcctgcag GTCGAC TTAGCACCGTCTCATCGG
210711	Fwd Up HR orf8052 EcoRI	Forward primer for the amplification of the upstream flanking region of orf8025. The primer contains a EcoRI site and overlaps with the suicide vector pEMG.	tagggataacagggtaatct GAATTC TAAGATCGTGGCGCTCC
210712	Rev Up HR orf8052	Reverse primer for the amplification of the upstream flanking region of orf8025. The primer overlaps with the 5′ of the downstream flanking region.	CAGGAGAGAGCGCAACACCG CGGAGGAGAGTAGGACAGA
210713	Fwd Dw HR orf8052	Forward primer for the amplification of the downstream flanking region of orf8025.	CGGTGTTGCGCTCTCTC
210714	Rev Dw HR orf8052 SalI	Reverse primer for the amplification of the downstream flanking region of orf5994-5512. The primer contains an SalI site and overlaps with the pEMG suicide vector.	agaagcttgcatgcctgcag GTCGAC AAGGCACTTCGGAACCATT
201104	amnB_1kb_Fw	Fwd primer for amplification of 1kb in the amnB gene for cloning into pJP with PclcA with In-Fusion kit	TAGGGATAACAGGGTAATCTGCTCTGTGCGGAACAAGGTTGGT
201105	amnB_1kb_Rev	Rev primer for amplification of 1kb in the amnB gene for cloning into pJP with PclcA with In-Fusion kit	GCTTGCATGCCTGCAGGTCGAGCGTGCATCCAAGTGAATGCA
